# Therapeutic properties of *Scutellaria baicalensis* in db/db mice evaluated using Connectivity Map and network pharmacology

**DOI:** 10.1038/srep41711

**Published:** 2017-01-31

**Authors:** Bu-Yeo Kim, Kwang Hoon Song, Chi-Yeon Lim, Su-In Cho

**Affiliations:** 1 Herbal Medicine Research Division, Korea Institute of Oriental Medicine, Daejeon, Republic of Korea; 2 Mibyeong Research Center, Korea Institute of Oriental Medicine, Daejeon, Republic of Korea; 3 College of Medicine, Dongguk University, Ilsan, Gyeonggi-do, Republic of Korea; 4 School of Korean Medicine, Pusan National University, Yangsan, Republic of Korea

## Abstract

We have reported that an extract of *Scutellaria baicalensis* (ESB) has effects against obesity and hypertriglyceridemia in type 2 diabetic animal model (db/db mouse). In the present study, we tried to explain the pharmacological effects of ESB by integrating gene expression information from db/db mouse liver with that of ESB-treated HepG2 hepatocellular carcinoma cells. Using Connectivity Map (cmap) analysis, we found an inverse relationship in the pharmaceutical profiles based on gene expression between db/db mouse liver and ESB-treated HepG2 cells. This inverse relationship between the two data sets was also observed for pathway activities. Functional network analysis showed that biological functions associated with diabetes and lipid metabolism were commonly enriched in both data sets. We also observed a similarity in distribution of cmap enrichment scores between db/db mouse liver and human diabetic liver, whereas there was an inverse pattern of cmap enrichment scores in human diabetic liver compared with ESB-treated HepG2 cells. This relationship might explain the pharmacological activities of ESB against db/db mouse and possible effectiveness of ESB against human diabetes. We expect that our approach using *in vitro* cell lines could be applied in predicting the pharmacological effectiveness of herbal drugs in *in vivo* systems.

In the development of novel drugs, including herbal drugs, cell-based *in vitro* screening systems are indispensable before the start of animal or clinical trials to reduce the cost and to save time in the screening of candidate drugs. Using established cell lines has many advantages for *in vitro* drug studies, such as the stable phenotype, availability, long lifespan, and ease of handling^1^. However, differences in the experimental conditions and inherent genetic backgrounds between *in vitro* and *in vivo* systems make it difficult to identify the targets that are common in both systems, and this can delay the development of pharmaceutically effective drugs. Therefore, it is important to be able to interpret the results from cell line experiments into a form that can be applicable to *in vivo* systems. Another obstacle, especially for the development of herbal drugs, is the complex nature of a drug’s chemical components and the difficulty in elucidating their main biological functions. Much of the research on herbal medicines has focused on identifying the biologically active components in herbal extracts.

The main components of *Scutellaria baicalensis* have been identified as baicalein, baicalin, wogonin, and so on^2^. The biological and pharmacological activities of individual chemical components contained in *S. baicalensis* have been shown to be involved in apoptosis and to have anti-inflammatory and antitumor properties^3^,^4^,^5^,^6^. We have previously shown that extracts of *S. baicalensis* (ESB) improved obesity and hypertriglyceridemia in type 2 diabetic animal model (db/db mouse)^7^. ESB has also been reported to be effective in treating diverse diseases through different pharmacological activities^8^,^9^,^10^,^11^, which suggests that the biological activities of ESB might not relate solely to the main ingredients. Moreover, ESB showed unique or enhanced pharmacological or biological characteristics that could not be explained by only a few major components^12^,^13^,^14^. Recent reports showed that the combination of pharmaceutically defined chemicals can cause synergistic effects of drugs and may even produce new pharmacological activities^15^,^16^. The complex interactions between components of drugs are more pronounced in herbal extracts, which generally comprise countless chemical components. Furthermore, identifying some key components of ESB or key biological targets could not be successfully accomplished on many occasions in herbal research. Therefore, in the present study, we attempted to evaluate the pharmacological effects of ESB in terms of multigene, multitarget and multifunctional effects. This recent trend in drug development and the complex nature of herbal extracts require a different approach from the classical approach that focuses on the direct relationship between individual targets and components.

Recently, the network pharmacological approach involving whole-genome-level information has been applied in herbal research in an attempt to identify multiple functional alternations induced by herbal extracts^17^,^18^. Moreover, successful development of pattern-matching algorithms based on non-parametric and rank-based strategies such as gene set enrichment analysis (GSEA)^19^ and their application in drug research using Connectivity Map (cmap) makes it possible to connect the changes in gene expression induced by drugs measured *in vitro* in cell lines with *in vivo* mechanisms and specific diseases^20^. Therefore, cmap may be useful for identifying the molecular mechanisms underlying the effects of herbal extracts and for predicting the pharmacological activities of herbal extracts, as we have previously suggested^21^,^22^.

In the present study, from cmap analysis, pharmaceutical profiles based on enrichment scores were inversely correlated between db/db mouse liver and ESB-treated HepG2 cells. Functional network analysis showed that biological functions such as diabetes- and lipid metabolism-associated functions were commonly enriched in both datasets. In addition, we observed a similarity in distribution of cmap enrichment scores between db/db mouse liver and human diabetic liver, whereas there was an inverse pattern of enrichment scores in human diabetic liver compared with ESB-treated HepG2 cells. We expect that cmap and a functional network approach using *in vitro* cell lines will be applicable in evaluating the pharmacological effectiveness of drugs with *in vivo* systems, especially for drugs comprising complex components that regulate the expression of many genes.

## Results

**Figure 1:**
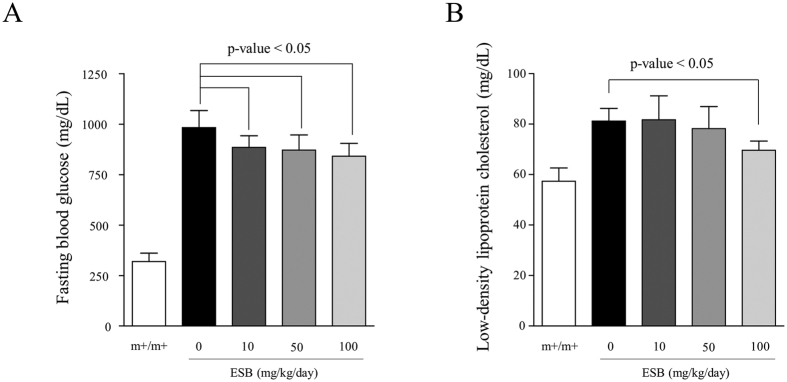
Effects of ESB on fasting glucose (**A**) and low-density lipoprotein cholesterol (**B**) concentrations in the blood of db/db mice. ESB was orally administered to db/db mice (10, 50 or 100 mg/kg body weight) daily for 4 weeks. Each experimental group included five animals. m+/m+ represents normal control mice of C57BLKS/J-m+/m+. The data were analyzed using Student’s *t* test.

**Figure 2:**
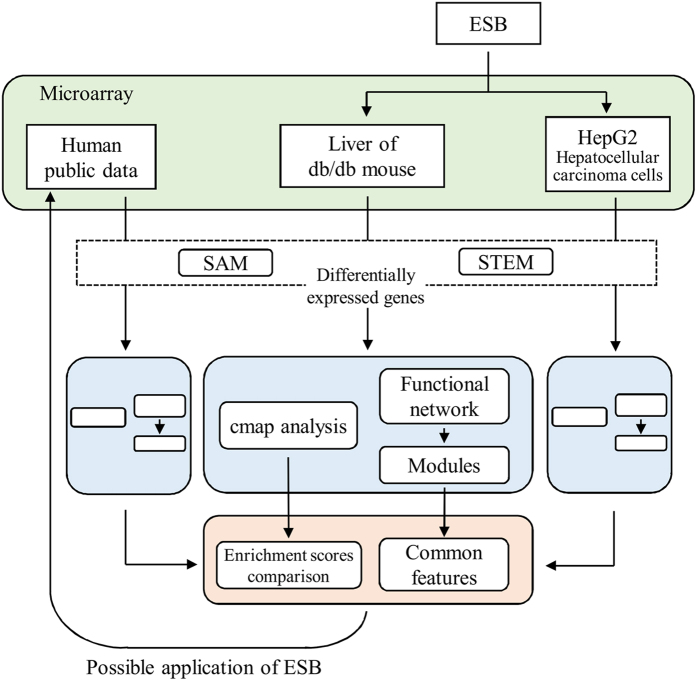
Schematic diagram of the analytical procedures. ESB was administered to db/db mice and HepG2 hepatocellular carcinoma cells. Differentially expressed genes in db/db mouse liver and ESB-treated HepG2 cells were queried into cmap to obtain the enrichment scores for 6100 reference chemical drugs archived in the cmap database to measure how closely the up and down tags of the queried gene signatures appeared similarly in the reference drugs. The resultant enrichment scores were compared between db/db mouse liver and ESB-treated HepG2 cells. Differentially expressed genes were also analyzed by functional module-based network analysis. Results of cmap and network analysis from db/db mouse liver and HepG2 cells were compared with those from samples of human diabetes for the evaluation of possible applications of ESB in humans.

We previously observed that ESB has effects against obesity and hypertriglyceridemia in the db/db mouse liver^7^. In the present study, we found that administration of ESB decreased the fasting blood glucose concentration (Fig. 1A) and low-density lipoprotein cholesterol concentration (Fig. 1B) in db/db mice. To extend these physiological changes, we examined the pharmacological effectiveness of ESB in terms of changes in the whole-genomic expression. The overall scheme of the present study is depicted in Fig. 2. First, microarray information from db/db mouse liver was entered into the cmap analysis to identify the pharmaceutical profile, which was then compared with that from ESB-treated HepG2 cells to evaluate the biological effects of ESB in db/db mice. Second, dynamic functional network analysis was performed to obtain common functional modules from db/db mouse liver and ESB-treated HepG2. We also used cmap to assess the similarities in the effects in the db/db mouse model and samples from patients with diabetes as a basis for possible applications of ESB in humans.

### Expression profile of genes regulated by ESB

**Figure 3:**
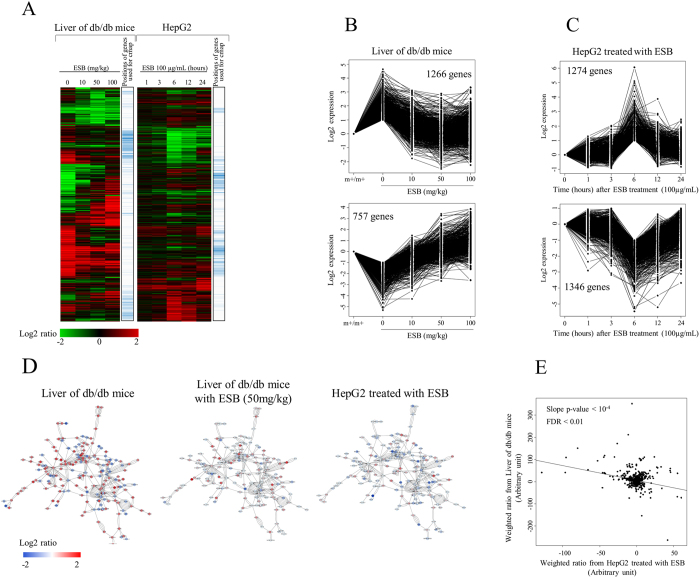
Effects of ESB on db/db mouse liver and HepG2 cells. (**A**) Expression patterns of genes were compared between db/db mouse liver given different doses of ESB and in HepG2 hepatocellular carcinoma cells exposed to ESB for different times. To identify the genes using the quantitative analysis of their expression, STEM was applied and the resultant ESB-responsive genes (FDR < 0.001) are plotted for (**B**) db/db mouse liver and (**C**) HepG2 cells. m+/m+ represents normal control mice of C57BLKS/J-m+/m+. (**D**) Differentially expressed genes in db/db mouse liver were mapped into the protein-interaction network database and the directly connected largest cluster was isolated. The levels of gene expression are depicted in colors as shown in the scale bar. (**E**) Log values for each differentially expressed gene in db/db mouse liver were multiplied by the degree value for each gene to give a weight according to the positional importance of the gene in the protein-interaction network. The weighted expression values were then plotted for db/db mouse liver and ESB-treated HepG2 cells.

We observed the overall expression pattern of genes in the livers of db/db mice and HepG2 cells treated with ESB. Mouse liver tissue was isolated after administration of ESB to mice at 10, 50, or 100 mg/kg body weight/day for 4 weeks. The overall pattern of gene expression in which a total of 8298 genes showing at least a 2-fold ratio variation when compared with that of the control was hierarchically clustered as shown in Fig. 3A. It is evident that the expression of 8298 genes was significantly changed in the livers of db/db mice compared with normal control mice. ESB administration altered the expression of these differentially regulated genes in db/db mouse liver and restored the levels of expression to the normal control level in a dose-dependent pattern. There were also newly regulated genes whose expression appeared in response to ESB. These effects of ESB resulted in clear differences from before to after ESB treatment and would make it difficult to evaluate the pharmacological effects of ESB at the individual gene level. We also measured the temporal pattern of gene expression in HepG2 cells after treatment with 100 μg/mL of ESB. As shown in Fig. 3A, gene expression changes peaked at 6 hours after ESB treatment. Comparative analysis of gene expression showed clear distinctions between liver tissue from db/db mice and HepG2 cells. The gene list shown in Fig. 3A is presented in Supplementary Fig. S1.

Then we applied quantitative analysis of gene expression using the short time series expression miner (STEM) program to select ESB-responsive genes with statistical significance^23^, through which we selected 6 and 8 patterns to be significant (FDR < 0.001) in db/db mice administered with ESB and HepG2 cells treated with ESB, respectively, as shown in Supplementary Fig. S2. Among these patterns of gene expression, we identified two types of inversely regulated patterns as major patterns in both datasets. In db/db mouse liver, initially 4087 and 3669 genes, at the 2-fold criterion, were up- and downregulated, respectively. Among these differentially expressed genes, 1266 (20.6%) and 757 (30.9%) genes were inversely regulated by ESB administration as identified by two representative patterns of reciprocal gene expression and shown in Fig. 3B. These reciprocally regulated genes by ESB administration in the livers of db/db mice could represent the therapeutic effects of ESB. The functional associations of these up- and downregulated genes were assessed using gene ontology (GO) and pathway enrichment analysis. Table 1 shows that, in db/db mouse liver, mRNA processing functions were associated with the upregulated genes and that cell signaling processes and sensory processes were associated with the downregulated genes. In the same analysis of ESB-treated HepG2 cells with the STEM program, we identified two types of reciprocally regulated genes whose expression peaked at 6 h after ESB treatment: 1274 and 1346 genes were up- and downregulated, respectively, at 6 hours after ESB treatment (Fig. 3C). Transcription-associated and RNA metabolism-associated biological functions were enriched in the genes downregulated by ESB, whereas no specific function except that the vesicular transport pathway was enriched in the genes upregulated by ESB in HepG2 cells (Table 1). ESB treatment for 6 hours was used as a representative condition for HepG2 cells in the following experiments.

Using interaction network analysis of the largest cluster composed of 180 nodes of differentially expressed genes in db/db mouse liver, we found that ESB administration recovered the expression of 128 core network genes to nearly normal control level. Interestingly, the core network-based pattern of gene expression in ESB-treated db/db mice was similar to that of ESB-treated HepG2 cells (Fig. 3D). Therefore, we measured the relationship based on core network gene expression between db/db mouse liver and ESB-treated HepG2 cells, in which inverse correlation (p-value < 10^−4^) was observed between two data sets (Fig. 3E); in the figure, degree values in the interaction network were used as weight for the calculation of weighted ratios of genes expression. This result implies that the pharmacological effect of ESB can be validated *in vitro* using HepG2 cells.

### Pathway activities

**Figure 4:**
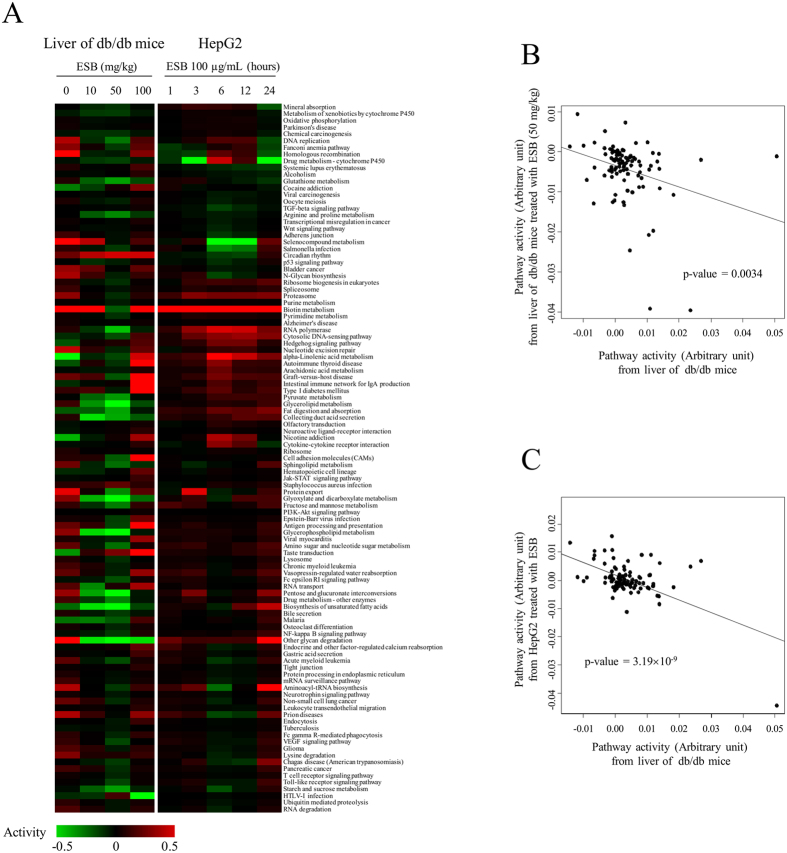
Effects of ESB on pathway activities. (**A**) For measurement of pathway activity, expression values of genes for each pathway compared with the normal control were log transformed and then linearly combined with a weight of –1 for genes acting as repressors. Statistically significant pathways (FDR < 0.01) were clustered according to the activity levels. The bar representing the activity value is presented in a color scale. (**B**) Plot of pathway activities in db/db mouse liver and the liver from ESB-treated (50 mg/kg) db/db mice. (**C**) Plot of pathway activities in db/db mouse liver and HepG2 cells treated with ESB for 6 hours.

We also observed the changes in pathway activities in an attempt to obtain biologically understandable changes induced by ESB treatment in db/db mouse liver and HepG2 cells. ESB administration (50 mg/kg) in db/db mice reversed many pathway activities altered in db/db mice liver tissue (Fig. 4A and B). However, ESB at 100 mg/kg did not show a similar effect on pathway activity (Fig. 4A). Interestingly, consistent with the result of expressional changes in network (Fig. 3E), pathways activities from db/db mouse liver show inverse relationship with those from the ESB-treated HepG2 cells (Supplementary Fig. S3). The quantitative measurement of pathway activity in the livers of db/db mice and ESB-treated HepG2 cells more clearly showed the inverse relationship (Fig. 4C). The recovery effect was most pronounced in the top 10 up- or downregulated pathways in db/db mouse liver (Supplementary Fig. S4). Analysis of the upper level of biological functions in the pathways showed that metabolism functions were upregulated whereas signaling functions were downregulated in db/db mouse liver compared with ESB-treated HepG2 cells (Supplementary Fig. S5).

### cmap analysis

**Figure 5:**
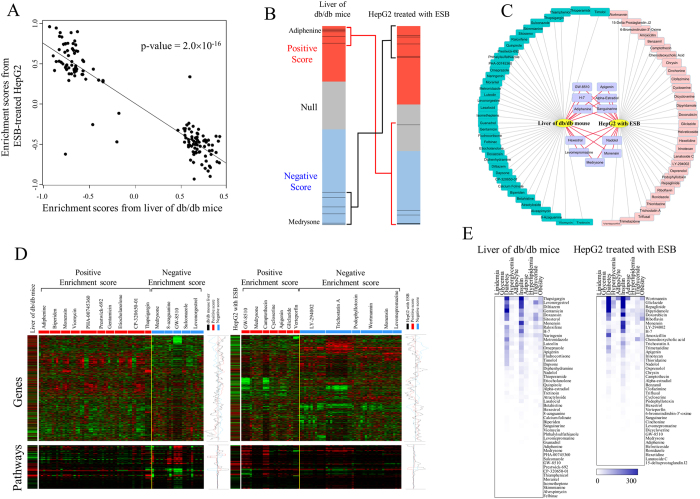
cmap analysis. Differentially expressed genes from db/db mouse liver and HepG2 cells treated with ESB were queried into cmap. The expression patterns of the genes queried are shown in Fig. 3A. (**A**) The enrichment scores of the top-ranked chemical drugs (permutated p-value < 0.05) resulting from cmap analysis of db/db mouse liver were compared with enrichment scores from HepG2 cells treated with ESB for 6 hours. (**B**) The position of the two top-ranked chemicals (adiphenine and medrysone) is plotted in the bar graph, which was constructed from 6100 individual chemicals ordered by their corresponding enrichment scores from +1 (top) to −1 (bottom). The red, gray, and blue colors represent the positive, null, and negative signs of the scores, respectively. Each line represents adiphenine and medrysone positioned differently according to different dosages and cell lines archived in the cmap database. (**C**) Comparison of chemical drugs obtained from the cmap analysis (permutated p-value < 0.01) of db/db mouse liver and HepG2 cells treated with ESB for 6 hours. Green, red, and purple node colors represent chemical drugs resulting from cmap analysis of db/db mouse liver, ESB-treated HepG2 cells, and both, respectively. (**D**) Profiles of gene expression and pathway activities for some highly ranked chemical drugs (permutated p-value < 0.001) from the cmap database. The average level of gene expression is shown according to the positive and negative scores. (**E**) A literature-mining approach was performed to measure the association between the cmap results and diabetes-related diseases. The following keywords were used for diabetes-related disease terms: diabetes, lipidemia, glycemia, hyperglycemia, hyperlipidemia, obesity, triglyceride, insulin, adipocyte, and adipose. For cmap results, the common names of chemical drugs with a permutated p-value < 0.01 were used as search keywords. The numbers of papers having keywords in the title or abstract published in 1990–2016 were obtained from the PubMed database. The numbers of papers are colored using the scale bar.

This inverse relationship between the livers of db/db mice and ESB-treated HepG2 cells prompted us to attempt a more systematic approach using cmap. The differentially expressed genes depicted in Fig. 3A were used in cmap analysis, in which 49 chemical drugs were selected to have the patterns of gene expression (permuted p-value < 0.01) that correlated with those in db/db mouse liver. The names of 49 drugs and their statistics are listed in Supplementary Table S1. A positive enrichment score represents a similar expression pattern and a negative enrichment score represents an inverse expression pattern of genes induced by chemical drugs compared with those in db/db mouse liver. We repeatedly obtained a chemical profile for the differentially expressed genes in ESB-treated HepG2 cells (Supplementary Table S1) and then compared the enrichment scores between db/db mouse liver and ESB-treated HepG2 cells. The plot of the enrichment scores of the top-ranked chemical drugs (permutated p-value < 0.05) in Fig. 5A shows an inverse relationship and a coefficient of −0.745 (p-value = 2.0×10^−16^), which is consistent with the results obtained from the analysis of the gene network and pathway activity (Figs 3E and 4C). Specifically, adiphenine and medrysone, the two top-ranked chemicals with enrichment scores of 0.915 and −0.827, respectively, in db/db mouse liver, were positioned inversely in ESB-treated HepG2 cells in which two chemicals had enrichment scores of −0.729 and 0.842, respectively (Fig. 5B). In addition to adiphenine and medrysone, nine chemicals were commonly selected with an inverse relationship (permuted p-value < 0.01) in the cmap analysis of db/db mouse liver and ESB-treated HepG2 cells (Fig. 5C).

The actual levels of gene expression and pathway activity regulated by the top selected chemicals (permuted p-value < 0.001) from the cmap database were compared with those of db/db mouse liver and ESB-treated HepG2 cells (Fig. 5D). In more detail, as shown in Supplementary Fig. S6, gene expression values (or activity values) from both datasets show significant positive or negative correlation (p < 0.01) compared with averaged values of gene expression (or activity values) from cmap samples of positive or negative enrichment scores, respectively. Only marginally significant relationship (p = 0.052) was found with cmap samples of positive enrichment scores in the pathway activity of ESB-treated HepG2 cells. This confirmed that the up- and downregulated genes and pathways in db/db mouse liver and ESB-treated HepG2 cells were also similarly or inversely regulated in positively or negatively associated samples in the cmap database, respectively.

For validation of our cmap approach, we measured the enrichment score profiles for 500 randomly selected drugs and compared them with those of db/db mouse liver and ESB-treated HepG2 cells. Supplementary Fig. S7 clearly demonstrates that drugs closely correlated with db/db mouse liver show an inverse correlation with ESB-treated HepG2 cells. This result indicates that drugs other than ESB could be successfully compared with db/db mouse liver using cmap analysis.

Finally, we used a keyword-based literature search to assess the association of the top selected chemicals (permuted p-value < 0.01) with the keywords related with diabetes-related diseases and conditions. The terms diabetes, hyperglycemia, insulin, obesity, and others were used as keywords for identification of papers in PubMed. As shown in Fig. 5E, chemicals obtained from the cmap analysis of db/db mouse liver and ESB-treated HepG2 cells have been reported to be associated with diabetes-related diseases or conditions. Using a randomized test, we found that results of a literature search using 49 and 41 chemical sets from db/db mouse liver and ESB-treated HepG2 cells, respectively, showed statistical significance (randomized test p-value < 0.05). In addition, by increasing the number of chemicals starting from the most referred chemical to a final chemical set of 49 and 41, we observed a statistically significant association (p < 0.05) of chemicals with diabetes-related diseases or conditions (Supplementary Fig. S8), which supports that our approach using cmap analysis may provide biologically relevant results in disease models. Molecular functions of the some top-listed drugs are listed in Supplementary Table S2.

### Modules identified in the dynamic functional network

**Figure 6:**
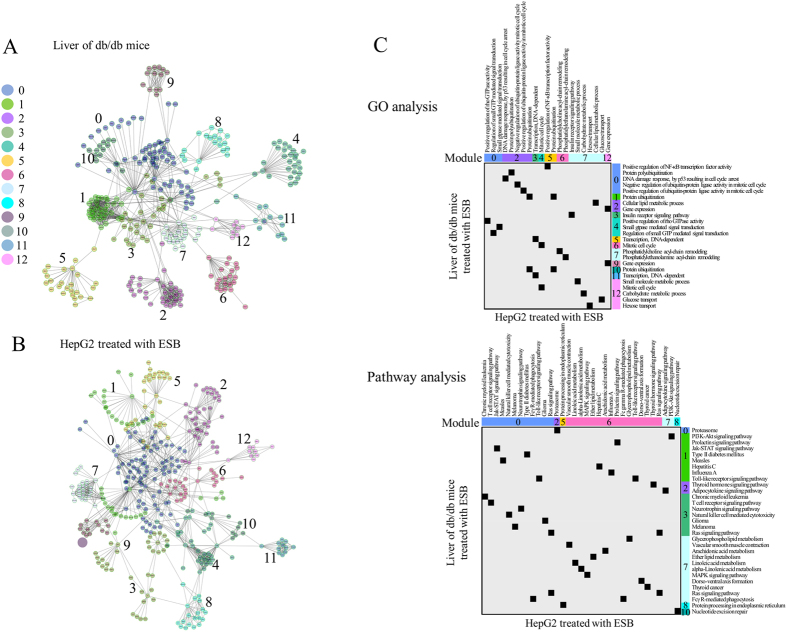
Functional network analysis. The functional interaction network was constructed from the gene expression patterns of (**A**) db/db mouse liver and (**B**) HepG2 cells treated with ESB by implementing the Reactome FI application. In total, 13 modules are shown in different colors as indicated from 0 to 12. (**C**) GO terms and pathways associated with each module (p < 0.01) were plotted for db/db mouse liver and HepG2 cells treated with ESB.

Because of the different patterns of gene expression and rarity of common genes between the samples, the approach based on single genes would not provide meaningful information about the relationships that exist commonly in db/db mouse liver and ESB-treated HepG2 cells. A gene set-based approach such as pathway analysis or cmap may be a good way to overcome this limitation of the single-gene approach. As another approach, we used dynamic functional network analysis of whole-gene expression. Figure 6A and B show the network modules obtained from db/db mouse liver and HepG2 cells treated with ESB, respectively. A list of gene nodes with network characteristics is presented in Supplementary Table S3. The biological functions (GO terms and pathways) associated with each module (FDR < 0.01) are listed in Supplementary Table S4. Despite the small number of common genes (22 genes) in these modules (see Supplementary Fig. S9 for a list of 22 common genes), many functions associated with modules were common between the two datasets with statistical significance (p < 0.01) (Fig. 6C). For example, signaling functions (NF-κB, G-protein-mediated signaling, Jak–STAT, MAPK signaling), cell cycle functions, and immune functions (chronic myeloid leukemia, Toll-like receptor, T-cell signaling, NK cell cytotoxicity, Fcγ R-mediated phagocytosis) were significantly enriched in both data sets. Metabolism and its related functions including protein ubiquitination, lipid metabolism, and carbohydrate metabolism were also enriched in both data sets. Interestingly, insulin receptor signaling, type II diabetes, and glucose metabolism were identified in both db/db mouse liver and HepG2 cells treated with ESB.

We also measured the involvement of individual core node genes (>10 degrees) in diabetes-related diseases or conditions using literature-based analysis, as described in the previous section. Interestingly, many core genes were reported to be associated with diabetes-related diseases or conditions, as shown in Supplementary Fig. S10. For example, eight genes (FABP4, SOCS3, EPO, GRB2, CNTF, PPARA, OSM and SOCS1) from db/db mouse liver and eight genes (RHOA, RAC1, STAT1, CBL, FYN, REL, RELA and NRF1) from ESB-treated HepG2 cells were highly referred (>100 times) in association with diabetes-related diseases or conditions. In addition to these genes, all genes, as a set, included in network modules were statistically significantly associated with diabetes-related diseases or conditions (randomized test p-value < 0.01). We also confirmed the functional relationship between db/db mouse liver and ESB-treated HepG2 cells by implementing GO term network analysis. As shown in Supplementary Fig. S11, GO terms enriched (p-value < 0.01, FDR < 0.1) in db/db mouse liver also included many of the differentially expressed genes in ESB-treated HepG2 cells. For example RNA processing functions, cell signaling processes, and cell sensory processes, which were enriched in db/db mouse liver, included ESB-responsive genes in HepG2 cells.

The results of the network analysis led us to conclude that diverse biological functions, including lipid and glucose metabolism, were commonly enriched as modules in both db/db mouse liver and ESB-treated HepG2 cells.

### Liver samples from diabetes patients

**Figure 7:**
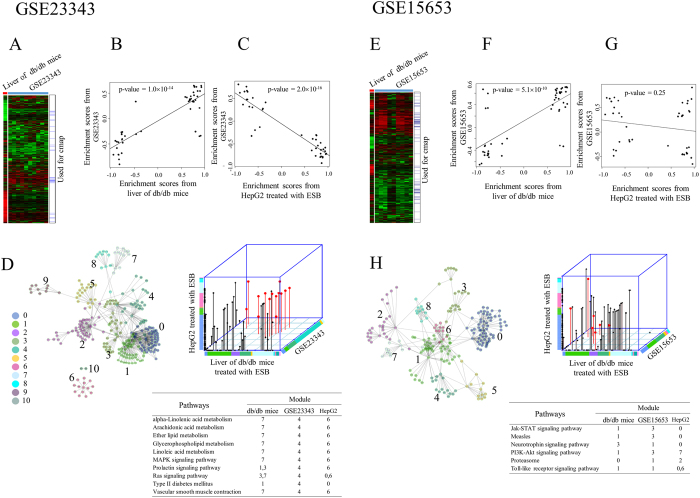
Analysis of human diabetic liver. Cmap and functional network analysis were performed using two public microarray data sets. Overall gene expression patterns are shown for (**A**) GSE23323 and (**E**) GSE15653 in which genes were hierarchically clustered according to ratio compared to the normal control. The positions of genes used for cmap analysis, which were selected from the SAM algorithm, are also indicated. Enrichment scores (permutated p-value < 0.05) resulting from the cmap analysis of GSE23323 were compared with those from (**B**) db/db mouse liver and (**C**) HepG2 cells treated with ESB. In same way, enrichment scores (permutated p-value < 0.05) resulting from cmap analysis of GSE15653 were compared with those from (**F**) db/db mouse liver and (**G**) HepG2 cells treated with ESB. Functional modules from the interaction network are presented in (**D**) for GSE23323 and (**H**) for GSE15653. Common pathways associated with each module (p < 0.01) from db/db mouse liver, HepG2 cells treated with ESB, and GSE23323 (or GSE15653) are shown in red and listed.

As a final experiment, we compared the differences in expression between db/db mouse liver and human diabetic liver as a basis for the possible application of ESB in the treatment of human diseases. We compared our results with the data in two public microarray data sets (GSE23343^24^ and GSE15653^25^), both of which relate to the human liver and diabetes. As in the previous case of db/db mouse liver and HepG2 cells, we could not obtain a significant number of genes commonly expressed in db/db mouse liver and the two data sets of liver from patients with diabetes (Fig. 7A and E). We then performed cmap analysis of differentially expressed genes (Supplementary Fig. S12) in disease samples contained in the two public data sets and then compared the distribution of enrichment scores between diabetic human liver, db/db mouse liver, and ESB-treated HepG2 cells. A plot of the enrichment scores shows significant correlation between db/db mouse liver and diabetic human liver (Fig. 7B and F), which suggests a similarity between liver tissues in db/db mice and diabetic humans. We also confirmed the negative correlation of enrichment scores between ESB-treated HepG2 cells and human diabetic liver (GSE23343) (Fig. 7C), although no clear correlation was observed in the GSE15653 sample (Fig. 7G). This pattern of cmap results is consistent with the previous pattern obtained from db/db mouse liver and ESB-treated HepG2 cells (Fig 5A). This result suggests that ESB has potential in the treatment of human diabetes.

We also measured pathway activities (FDR < 0.01) in human diabetic liver and compared these with those of db/db mouse liver and ESB-treated HepG2 cells. As in the previous case (Fig. 4B and C), positive or negative relationship of human diabetic liver were observed with db/db mice liver or HepG2 treated with ESB, respectively (Supplementary Fig. S13).

Functional network analysis identified eleven and nine dynamic functional modules in the GSE23343 and GSE15653 datasets, respectively. Pathways associated with each module (FDR < 0.01) showed that the lipid metabolism pathways and diabetes pathway were commonly enriched in the human diabetic liver (GSE23343), db/db mouse liver, and ESB-treated HepG2 cells (Fig. 7D). However, we could not obtain similar functional involvement in the other human diabetic liver data set (GSE15653), in which the signaling pathways were enriched significantly (Fig. 7H). Similar functional associations of these modules were also observed in the GO terms, in which lipid-associated terms were commonly enriched in human diabetic liver (GSE23343), db/db mouse liver, and ESB-treated HepG2 cells (Supplementary Fig. S14).

From the analysis of human diabetic samples, we found that the liver in diabetic humans has many properties in common with db/db mouse liver and shows the inverse relationship with ESB-treated HepG2 cells. This suggests that ESB has potential in the treatment of human diabetes.

## Discussion

The scientific basis of using *in vitro* systems for drug development is that biological changes, especially changes in gene expression induced by candidate drugs in cell line-based systems, can be reproduced in *in vivo* systems. However, considering the different biological conditions between *in vitro* cells and living organisms, it is expected that gene expression changes would differ between cell line-based systems and *in vivo* systems. In the present study, we tried to link gene expression in the HepG2 cancer cell line with that of a db/db mouse model to identify the common biological changes induced by ESB and to try to predict the pharmaceutical effects of ESB on human diabetes.

The responses of HepG2 cells to ESB, especially the effects on cell growth and apoptosis, have been reported. Ye *et al*. reported that treatment with 200 μg/mL of *S. baicalensis* induced strong inhibition of HepG2 cell growth and decreases in expression of cell cycle-related proteins such as p53, MAPK1, CDC25B, CDC25C, EGFR, and Hif-2α^5^. In addition to growth inhibition^3^,^6^, metastasis inhibition by *S. baicalensis* was also measured in HepG2 cells^11^. The reports mentioned above and other studies on different cancer cell lines^26^,^27^ are the basis for considering that *S. baicalensis* is a potential anti-cancer agent. However, in addition to its anti-proliferative activity, diverse effects of *S. baicalensis* have also been reported such as anti-allergic, antioxidant, anti-inflammatory, and neuroprotective effects^28^. These diverse effects of *S. baicalensis* should depend on the dosage, treatment duration, and cell type used in each experiment. For example, in our study, 100 μg/mL of ESB did not have a growth-inhibitory effect within 24 hours, whereas other studies have reported anti-growth effects of *S. baicalensis* at much higher concentration in HepG2 cells^3^,^6^. Therefore, it is clear that the effects of *S. baicalensis* cannot be revealed simply by examining one aspect of the biological responses. Moreover the drug causes changes in the expression of many diverse genes, as shown in Fig. 3. Therefore, it is important to use as many responsive genes against external or internal stimuli as possible and to study their relationships to infer more precisely the changes in biological functions.

By applying thousands of differentially expressed genes in the cmap analysis, we identified inverse relationships in the pharmaceutical profiling patterns between db/db mouse liver and ESB-treated HepG2 cells. But for the possible prediction of pharmacological effectiveness of ESB, there are some limitations to our approach. Most of all, there are differences in the biological systems used in the present analysis. First, we should consider the difference between the *in vitro* cell line and *in vivo* model. The cmap database is constructed based on *in vitro* cancer cell lines. Therefore, our gene expression data from HepG2 cells could be directly compared with those of the cmap database, but implementing gene expression information obtained from liver tissue of mouse into cmap could possibly result in error. Second, species-specific gene expression should also be considered. Although we matched mouse gene symbols with human gene symbols using a gene orthology database, it is certain that gene-regulated systems in mice would be different to those of human cells. Third, the cmap database consists of cancer-cell-based microarray information. Four different types of cancer cells (MCF7, PC3, SKMEL5 and HL60) have been used for the identification of drug-responsive genes in the cmap database. Although it would not be a critical problem when genes from only cancer cells are applied to the cmap analysis, as in our previous report^21^, implementing cmap with the results of genes from non-cancerous samples should be interpreted carefully. We also confirmed the actual correlations in the gene expression patterns between the top selected cmap samples (permuted p-value < 0.01) and our samples (Fig. 5D).

Because it is hard to obtain biological information about the molecular actions of ESB by observing individual genes, we used gene set-based approaches such as pathway activities and dynamic functional network analysis. As shown in Fig. 4 and Supplementary Fig. S3, many pathways with diverse biological functions had inverse relationships between db/db mouse liver and ESB-treated HepG2 cells, which may at least partially explain the results of the cmap analysis. Functional classification of pathways showed more clearly that metabolism-related pathways were activated primarily in db/db mouse liver, whereas other pathways, such as those involved in signaling or diseases, were downregulated in comparison with ESB-treated HepG2 cells (Supplementary Fig. S5). Interestingly, the linkage between metabolism and signaling processes is becoming increasingly evident in diverse cellular conditions in which protein modification such as glycosylation, acetylation, and phosphorylation is thought to play an important role in the reciprocal regulation of metabolism and signaling processes^29^,^30^.

Whereas this reciprocal relationship between metabolism and signaling processes may indicate a global regulatory mechanism, the interconnections of biological functions in response to ESB may show more detail of the functional network structure (Fig. 6). Despite the fact that few common genes were identified (Supplementary Fig. S9), we found that many common biological functions such as insulin receptor signaling, type II diabetes, lipid metabolism, and glucose metabolism were enriched in modules from db/db mouse liver and HepG2 cells treated with ESB. From these results, we conclude that diverse interrelated functions were identified in both the db/db mouse liver and ESB-treated HepG2 cells and that these common patterns may be helpful for predicting *in vivo* pharmacological effects of ESB using *in vitro* experiments.

In addition to the network module-based evidence, we also observed that core node (>10 degrees) genes enriched in db/db mouse liver and ESB-treated HepG2 cells are associated with diabetes-related diseases or conditions. For example, in the network from ESB-treated HepG2 cells, *RHOA* (28 degrees in the network structure), which encodes the small GTPase RhoA, is involved in the mediation of numerous signaling processes related to diabetes and hyperglycemia^31^. Moreover, *RHOA* is involved in various pathological conditions occurring in diabetic complications such as retinopathy^32^, microvascular endothelial dysfunction^33^, and venous impairment^34^. *RAC1* (36 degrees) also plays a pivotal role in cardiomyocyte apoptosis during hyperglycemia^35^. *STAT1* represents another core node (17 degrees) and is involved in the regulation of pancreatic beta-cell apoptosis and islet inflammation^36^. The CBL-dependent insulin-signaling pathway is involved in regulating glucose uptake^37^,^38^. In addition to the abovementioned genes, many core nodes (>10 degrees) are associated with diabetes-related diseases or conditions. These relationships support the idea that individual core genes or modules of genes identified using network analysis may link *in vitro* changes in ESB-treated HepG2 cells with diseases and conditions as in the case for cmap analysis reported here.

We finally investigated whether our approach using cmap and functional network analysis of db/db mouse liver and HepG2 cells could be confirmed in data sets of human diabetes. Similar with the results of the mouse and cell line experiments, enrichment scores from livers of diabetic humans correlated positively with those of db/db mouse liver but inversely with those of ESB-treated HepG2 cells. The lipid-associated metabolism and diabetes pathways were commonly enriched among all three data sets—human diabetic liver, db/db mouse liver, and ESB-treated HepG2 cells. These results support the idea that ESB may be effective in the treatment of human diabetes. Although we have focused here mainly on the technical aspects for the explanation of the inverse relationship between ESB-treated HepG2 cells and db/db mice, if we can elucidate more precisely the biological and cellular changes occurring in both systems, we could develop a more accurate evaluation model for measuring the pharmacological effectiveness of ESB in *in vitro* systems.

In conclusion, using cmap analysis, we observed that the enrichment scores of ESB-treated HepG2 cells correlated inversely with those of db/db mouse liver. Network analyses supported the functional association between these two samples, which might explain the pharmacological activities of ESB at the molecular level. We hope that our approach using *in vitro* cell lines will be applicable in predicting the pharmacological effectiveness of drugs in *in vivo* systems, especially for drugs comprising complex components that regulate the expression of many genes.

## Methods

### Preparation of *S. baicalensis*

*Scutellaria baicalensis* was purchased from the Hyundai Herbal Medicine Company (Youngchun, Republic of Korea). According to the traditional method for preparation of this herbal medicine, 300 g of *S. baicalensis* was boiled in 6 L of water for 3 hours. The crude extract was filtered and lyophilized in a freeze-dryer, as described previously^7^. A final 115 g dry weight of ESB was obtained, which corresponded to 38.3% yield. The ESB was kept in that state and dissolved in deionized distilled water shortly before its administration to the model mice and HepG2 cells. High-performance liquid chromatography analysis of ESB was described in detail in a previous report in which we confirmed the presence of representative components of *S. baicalensis* such as baicalin, baicalein, and wogonin^7^.

### Animal experiments

Six-week-old male C57BLKS/J-m+/m+ (n = 5) and C57BLKS/J-db/db (n = 15) mice were obtained from Central Lab. Animal, Inc. (Seoul, Republic of Korea). C57BLKS/J-m+/m+ mice were used as normal controls and C57BLKS/J-db/db mice were used as disease model mice in the experiments. All mice were housed individually in polycarbonate cages at a controlled temperature of 23 ± 3 °C with 60% humidity and a 12-hour light/dark cycle. Standard chow diet (catalog No 6112, Central Lab. Animal, Inc.) and water were provided *ad libitum* throughout the experiment. After 2 weeks of acclimation under these conditions, the mice were randomly divided into five groups of five mice each: (1) vehicle-treated C57BLKS/J-m+/m+ mice (n = 5), (2) vehicle-treated C57BLKS/J-db/db (n = 5), (3) C57BLKS/J-db/db mice treated with ESB (10 mg/kg body weight, n = 5), (4) C57BLKS/J-db/db mice treated with ESB (50 mg/kg body weight, n = 5), and C57BLKS/J-db/db mice treated with ESB (100 mg/kg body weight, n = 5). Vehicle (water) or ESB dissolved in water (10, 50, or 100 mg/kg body weight) was administrated orally daily for 4 weeks. All mice were fasted overnight and anesthetized using 5% isoflurane before sacrifice. All efforts were made to minimize pain during sacrifice. Blood from anesthetized mice was collected through cardiac puncture, and the serum was separated immediately from the blood and stored at −80 °C until use. The livers were excised, rinsed with phosphate-buffered saline, snap-frozen in liquid nitrogen, and stored at –80 °C until use.

All experimental procedures were approved by the Institutional Animal Care and Use Committee of the Korea Institute of Oriental Medicine and were performed under strict accordance with the recommendations in the Guide for the Care and Use of Laboratory Animals at the Korea Institute of Oriental Medicine.

### Cell experiments

The HepG2 human hepatocellular carcinoma cell line was obtained from the American Type Culture Collection (HB-8065; Manassas, VA, USA). Authentication of the HepG2 cell line was confirmed using a short tandem repeat analysis by Korean Cell Line Bank (Seoul National University College of Medicine, Seoul, Republic of Korea). HepG2 cells were maintained in a 1:1 mixture of Dulbecco’s modified Eagle’s medium (DMEM) and F-12 (50:50; DMEM/F12, Invitrogen, Carlsbad, CA, USA) supplemented with 10% (v/v) fetal bovine serum (FBS), 100 U/mL penicillin, and 100 μg/mL streptomycin in 5% CO_2_ in humidified air at 37 °C. Cells were used when they reached 75% confluence. After 24 hours of starvation in DMEM/F12 supplemented with 0.5% FBS, cells were exposed to ESB at 100 μg/mL for 1–24 hours. All materials used for the cell cultures were purchased from Invitrogen (Carlsbad, CA, USA).

### Microarray experiment

Total RNA was purified from the liver tissue and HepG2 cells using an RNeasy kit according to the manufacturer’s instructions (Qiagen, Hilden, Germany). The quality of isolated RNA was measured using Agilent’s Bioanalyzer 2100 RNA Nano kit (Agilent Technologies, Santa Clara, CA, USA). Only those samples with an RNA integrity number (RIN) >7.0 were included in the microarray analysis. For liver tissues, an equal amount of isolated RNAs from five mice per experimental group was pooled before the microarray analysis to eliminate individual variability. The isolated RNA was amplified and labeled using a Low RNA Input Linear Amplification kit PLUS following the manufacturer’s instructions (Agilent Technologies) and then hybridized to a microarray (Agilent Mouse Whole Mouse Genome 44 K for mouse liver tissues and Agilent Human Whole Genome 44 K for HepG2 cells; Agilent Technologies) in accordance with the manufacturer’s instructions. The arrays were scanned using an Agilent DNA Microarray Scanner, and the signal intensities were extracted using Agilent Feature Extraction Software (Agilent Technologies). The dataset is available online at the Gene Expression Omnibus ( http://www.ncbi.nlm.nih.gov/geo) under the ID number GSE84783.

### Microarray analyses

Only probes with signal intensities 1.4-fold higher than the local background were selected as significant probes, as described previously^21^,^22^. The local background subtracted signal intensities were then normalized using the quantile method to adjust for variability within samples and between samples^39^. Genes showing at least a 2-fold ratio variation when compared with those of the control were hierarchically based on expression level using the average linkage method of the Gene Cluster 3.0 program and visualized using the TreeView program ( http://www.eisenlab.org/eisen/). To identify dose or time dependently expressed genes, the short time series expression miner (STEM) program was used in which the statistical significance of the resultant expression patterns was measured as false discovery rates (FDRs) using 1,000 random permutations^23^. Mouse gene symbols were compared with human gene symbols using the gene orthology database maintained by the Jackson Laboratory ( http://www.informatics.jax.org).

### cmap analysis

Differentially expressed genes in the livers of ESB-treated db/db mice and ESB-responsive genes in HepG2 cells isolated by the STEM program were queried into cmap 02 ( http://www.broadinstitute.org/cmap/), in which rank-based pattern-matching algorithms are implemented for the similarity measurement of the input query signature from the reference microarray data comprising 6100 samples treated with diverse pharmaceutical substances^20^. In brief, the query signatures were compared with the rank-ordered list of reference microarray data to yield enrichment scores, which measure how closely the “up’ or “down” tags of the query signatures appear in the top or bottom of each reference microarray, respectively. The statistical significance was then computed based on random permutations.

For validation of the cmap-based approach, 500 chemicals were randomly chosen from the cmap database and analysed by cmap for enrichment scores. We used the top 1000 differentially expressed genes for each random chemical. The distribution of enrichment scores for random chemicals was compared with that from db/db mouse liver and ESB-treated HepG2 cells. Correlation coefficients obtained from db/db mouse liver and ESB-treated HepG2 cells were plotted for random chemicals.

### Gene ontology (GO) and pathway analyses

The enrichment of GO terms and pathways were measured using the Functional Annotation Tool of Database for Annotation, Visualization and Integrated Discovery (DAVID) for the differentially expressed genes. Statistical significance was calculated using the modified Fisher’s exact test and adjusted using the Benjamini–Hochberg procedure^40^. Pathway activity was measured using the expression values of all genes included in each pathway to account for the accumulative effect of all the genes in a pathway^41^,^42^. Briefly, the expression values of genes in the experimental groups were transformed to a ratio relative to the control sample, log-transformed, and then linearly combined with a weight of –1 for genes acting as repressors in each pathway. For identification of repressors, we manually selected proteins acting as inhibitors in the process of signal transduction for each pathway. The measured value was normalized by dividing it by the size of the pathway. The statistical significance of the measured values was calculated using permutations of random iterations of 1000. The pathway information used for enrichment and activity measurement was obtained from the Kyoto Encyclopedia of Genes and Genomes database (KEGG, http://www.genome.jp/kegg/).

### Network-based drug efficacy

Differentially expressed genes in the liver of db/db mice were mapped into a protein interaction network, and the largest cluster comprising directly connected nodes was isolated. The log ratio relative to the control sample of each gene in the cluster was multiplied by a degree to give a weighted value, which reflects the positional importance of that gene in the protein interaction network. These values for all genes in the cluster were linearly combined to give weighted ratios. The statistical significance of weighted ratios in the correlation plot was measured by permutations (1000 times) of randomly selected genes. Information for the interaction network was obtained from BioGRID (version 3.4)^43^ and visualized using Cytoscape^44^.

### Functional network analyses

Functional network structure was constructed using the Reactome FI network Cytoscape plugin application ( http://www.reactome.org/)^45^. Specifically, differentially expressed genes with a fold ratio >2 or < 0.5 compared to the control level in at least one sample were queried into Reactome FI, whose database comprises information about protein–protein interactions, gene coexpression, protein domain interactions, and text-mined protein interactions. Modules comprising subsets of correlated genes were identified using the Markov Cluster Algorithm with Pearson correlation coefficients of 0.8 and a default setting for other parameters. The statistical significance of overlapping of modules from network analysis was measured by applying a hypergeometric test in which population sets for GO and pathway analysis were extracted from the Reactome FI and KEGG pathway databases.

Functional connections based on gene expression between the livers of ESB-treated db/db mice and ESB-treated HepG2 cells were identified using the Enrichment Map Plugin for Cytoscape ( http://baderlab.org/Software/EnrichmentMap/)^46^. For node parameters, we used FDR < 0.01 to select significantly enriched GO terms from the DAVID analysis of the microarray data. For edge (gene set relationship) parameters, a similarity cut-off of 0.5 using Overlap Coefficient (default setting) was used. To obtain the relationship (edge width) of GO terms with differentially expressed genes from the other dataset, a hypergeometric test with 0.01 cut-off was performed for the determination of edge weight in the post-analysis of the Enrichment Map.

### Keywords-based literature-mining analysis

A literature-mining approach was used to measure the association between the cmap results and diabetes-related diseases. The following keywords were used for diabetes-related disease terms: diabetes, lipidemia, glycemia, hyperglycemia, hyperlipidemia, obesity, triglyceride, insulin, adipocyte and adipose. For cmap results, the common names of chemical drugs with a permutated p-value < 0.01 were used as search keywords. The numbers of papers with keywords in the title or abstract published in 1990–2016 were collectively obtained from the PubMed database using the PubMatrix algorithm^47^. The numbers of papers were indicated by different colours on a scale bar. For a random permutation-based test, we used all the chemicals (1309 chemicals) present in the cmap database to search for papers with keywords connected to diabetes-related diseases and conditions. We then changed the referred numbers of papers to 0 (absence of reference paper) and 1 (presence of reference paper) to eliminate bias induced by a few heavily referred chemicals. We then compared the total sum of the original dataset with the same-sized randomly selected dataset repeatedly 1000 times. The relative positon of the sum of the original dataset among those of the randomized test sets was measured as a ratio and used as a statistic (randomized test p-value). We also measured this value by increasing the number of chemicals included in the original dataset starting from the most referred chemical. We also measured the association between genes and diabetes-related diseases using the keywords for diabetes-related disease terms. For validation of the gene-based search, we compared the results of the original genes with those of randomly selected genes using the method described above.

### Public microarray data

Two public microarray data sets (GSE23343^24^ and GSE15653^25^) deposited in Gene Expression Omnibus ( http://www.ncbi.nlm.nih.gov/geo) were used in this analysis. The two public microarray data sets related to human liver samples from patients with type 2 diabetes were used. CEL files were imported into the Affy R package and normalized using the Robust Multiarray Average (RMA) algorithm. To identify the genes used as the input tags in the cmap database, Significance Analysis of Microarrays (SAM)^48^ was performed under the two-class response type (normal (n = 7) vs. type 2 diabetes (n = 10) in GSE23343 and normal (n = 5) vs. obese diabetes (n = 9) in GSE15653). For GSE23323 and GSE15653, SAM scores of absolute values of 2.0 and 4.0 were used for threshold values, which corresponded to an FDR < 0.3 and FDR < 0.05, respectively (Supplementary Fig. S12).

## Additional Information

**How to cite this article**: Kim, B.-Y. *et al*. Therapeutic properties of *Scutellaria baicalensis* in db/db mice evaluated using Connectivity Map and network pharmacology. *Sci. Rep.* **7**, 41711; doi: 10.1038/srep41711 (2017).

**Publisher's note:** Springer Nature remains neutral with regard to jurisdictional claims in published maps and institutional affiliations.

## Supplementary Material

Supplementary Tables and Figures

## Figures and Tables

**Table 1:** GO terms and pathways (FDR < 0.01) associated with differentially expressed genes in db/db mouse liver and ESB-treated HepG2 cells.

ID	Up-regulated genes				Down-regulated genes		
	GO term	p-value^1^	FDR^2^	ID	GO term	p-value	FDR
**Enriched GO terms**							
**Liver of db/db mice**							
GO:0006396	RNA processing	4.68E-07	1.72E-03	GO:0007186	G-protein coupled receptor protein signaling pathway	1.61E-18	5.59E-15
GO:0006397	mRNA processing	7.46E-07	1.37E-03	GO:0007600	Sensory perception	2.92E-16	5.79E-13
GO:0016071	mRNA metabolic process	8.66E-07	1.06E-03	GO:0050890	Cognition	1.10E-15	1.29E-12
GO:0006350	Transcription	8.55E-06	7.84E-03	GO:0007606	Sensory perception of chemical stimulus	7.81E-15	6.76E-12
				GO:0050877	Neurological system process	4.04E-14	2.81E-11
				GO:0007608	Sensory perception of smell	1.73E-13	1.01E-10
				GO:0007166	Cell surface receptor linked signal transduction	5.86E-13	2.91E-10
ESB-responsive genes in up-regulated genes				ESB-responsive genes in down-regulated genes			
GO:0007167	Enzyme linked receptor protein signaling pathway	1.07E-06	3.05E-03	GO:0051726	Regulation of cell cycle	3.94E-05	4.45E-03
GO:0006397	mRNA processing	2.11E-06	3.00E-03	GO:0044057	Regulation of system process	5.40E-05	4.89E-03
GO:0016071	mRNA metabolic process	4.12E-06	3.90E-03	GO:0060341	Regulation of cellular localization	6.81E-05	4.96E-03
GO:0006396	RNA processing	5.32E-06	3.78E-03	GO:0050804	Regulation of synaptic transmission	7.36E-05	5.00E-03
				GO:0043068	Positive regulation of programmed cell death	1.43E-04	1.00E-02
**ESB-treated HepG2**							
Up-regulated genes				Down-regulated genes			
ID	GO term	p-value	FDR	ID	GO term	p-value	FDR
NS	NS	NS	NS	GO:0035295	Tube development	7.32E-06	2.30E-04
				GO:0045449	Regulation of transcription	1.08E-05	1.70E-03
				GO:0051252	Regulation of RNA metabolic process	1.80E-04	6.16E-03
				GO:0051253	Negative regulation of RNA metabolic process	1.90E-04	5.87E-03
**Enriched pathways**							
**Liver of db/db mice**							
Up-regulated genes				Down-regulated genes			
ID	Pathway	p-value	FDR	ID	Pathway	p-value	FDR
mmu03040	Spliceosome	1.47E-04	2.74E-03	mmu04740	Olfactory transduction	1.25E-12	2.25E-10
				mmu05320	Autoimmune thyroid disease	5.92E-04	5.19E-03
ESB-responsive genes in up-regulated genes				ESB-responsive genes in down-regulated genes			
mmu03040	Spliceosome	6.87E-05	1.21E-03	mmu04080	Neuroactive ligand-receptor interaction	2.57E-04	3.80E-03
**ESB-treated HepG2**							
Up-regulated genes				Down-regulated genes			
hsa04130	SNARE interactions in vesicular transport	2.23E-04	3.56E-03	NS	NS	NS	NS

^1^p-values were calculated using the Fischer’s test.

FDR corrections were calculated using the Benjamini-Hochberg procedure.
