# Supplementary material for: Therapeutic properties of Scutellaria baicalensis in db/db mice evaluated using Connectivity Map and network pharmacology
Source: Sci Rep. 2017 Jan 31;7:41711. doi: 10.1038/srep41711 (PMC5282526; doi:10.1038/srep41711)
Supplement: Supplementary Tables and Figures [file srep41711-s1.pdf]

Supplementary information

Therapeutic properties of *Scutellaria baicalensis* in db/db mice evaluated using Connectivity Map and network pharmacology

Bu-Yeo Kim [1,†,*], Kwang Hoon Song [2,†], Chi-Yeon Lim [3], Su-In Cho [4]

[1] Herbal Medicine Research Division, Korea Institute of Oriental Medicine, Daejeon, Republic of Korea

[2] Mibyeong Research Center, Korea Institute of Oriental Medicine, Daejeon, Republic of Korea

[3] College of Medicine, Dongguk University, Ilsan, Gyeonggi-do, Republic of Korea

[4] School of Korean Medicine, Pusan National University, Yangsan, Republic of Korea

[†] Co-first author

[*] Correspondence: Bu-Yeo Kim, PhD

Herbal Medicine Research Division, Korea Institute of Oriental Medicine,

1672 Yuseong-daero, Yuseong-gu, Daejeon, 34054, Republic of Korea

Phone: +82 42 868 9401, Fax: + 82 42 868 9541

Email: buykim@kiom.re.k

Supplementary Table S1. cmap analysis

Liver of db/db mice					HepG2 treated with ESB		
Rank[1]	Name[2]	Enrichment score[3]	p-value[4]		Name	Enrichment score	p-value
1	Adiphenine	0.915	0		Gw-8510	0.943	0
2	Medrysone	-0.827	0.00002		Ly-294002	-0.47	0
3	Biperiden	0.905	0.00004		Trichostatin A	-0.344	0
4	8-azaguanine	-0.896	0.0001		Podophyllotoxin	-0.931	0.00002
5	Monensin	0.793	0.00016		Medrysone	0.842	0.00002
6	Viomycin	0.889	0.00034		Wortmannin	-0.571	0.00002
7	Pha-00745360	0.68	0.0004		Monensin	-0.831	0.00006
8	Gw-8510	-0.862	0.0005		Camptothecin	0.954	0.0001
9	Sulconazole	-0.862	0.0005		Cycloserine	0.901	0.0001
10	Prestwick-692	0.878	0.00052		Apigenin	0.888	0.00016
11	Gentamicin	0.878	0.00052		Gliclazide	0.886	0.00018
12	Etiocholanolone	0.744	0.00054		Levomepromazine	-0.898	0.0002
13	Levonorgestrel	-0.748	0.0007		Verteporfin	0.937	0.00042
14	Cp-320650-01	0.65	0.00086		6-bromoindirubin-3'-oxime	0.672	0.00108
15	Thapsigargin	0.917	0.00096		Thioridazine	-0.402	0.00179
16	Alpha-estradiol	-0.46	0.0011		Sanguinarine	0.963	0.00239
17	Omeprazole	-0.832	0.00117		Clofazimine	-0.735	0.00274
18	Timolol	0.837	0.00123		Adiphenine	-0.729	0.00304
19	Doxazosin	-0.83	0.00127		Alpha-estradiol	0.433	0.00309
20	Naringenin	0.835	0.00133		Nadolol	-0.798	0.00328
21	Thiamphenicol	0.761	0.00146		Riboflavin	0.783	0.00412
22	Atractyloside	0.751	0.00176		Irinotecan	0.864	0.00473
23	Apigenin	-0.821	0.00179		Dipyridamole	0.649	0.00544
24	Phthalylsulfathiazole	-0.756	0.00206		Helveticoside	-0.645	0.00554
25	Fludrocortisone	0.611	0.00221		Dicycloverine	-0.695	0.00575
26	Levomepromazine	0.813	0.00229		Doxorubicin	0.854	0.00597
27	Luteolin	-0.811	0.00241		Ronidazole	0.851	0.00619
28	Metronidazole	0.739	0.00246		Chenodeoxycholic acid	-0.758	0.00696
29	Morantel	-0.742	0.00264		Oxprenolol	0.752	0.00722
30	Quinpirole	0.795	0.00348		Hexestrol	0.751	0.00742
31	Diphenhydramine	0.703	0.00505		Triflusal	0.843	0.00755
32	Isometheptene	0.774	0.00533		H-7	0.748	0.00774
33	Tretinoin	-0.354	0.00546		Hexetidine	-0.749	0.00794
34	Sitosterol	-0.769	0.00553		Repaglinide	0.744	0.00804
35	Lasalocid	0.77	0.00567		Cinchonine	-0.746	0.00822
36	Thioperamide	0.695	0.00575		Lanatoside C	-0.627	0.0085
37	H-7	-0.764	0.00585		Amoxicillin	0.742	0.00855
38	Skimmianine	-0.761	0.00621		15-delta prostaglandin J2	-0.409	0.00859
39	Hexestrol	-0.761	0.00625		Chrysin	0.835	0.00895
40	Diltiazem	-0.695	0.00653		Trimetazidine	0.735	0.00975
41	Nadolol	0.759	0.00688		Benzamil	-0.619	0.00991
42	Alvespimycin	-0.467	0.0069				
43	Felbinac	0.758	0.00696				
44	Guanadrel	0.686	0.00711				
45	Betahistine	-0.751	0.0074				
46	Sanguinarine	-0.937	0.00769				
47	Calcium folinate	0.673	0.00879				
48	Dapsone	0.672	0.00881				
49	Raloxifene	0.577	0.0099				

[1] Rank is based upon estimates of the likelihood that the enrichment of a set of instances in the ordered list of all instances for that result would be observed by chance in cmap database.

[2] Name given to a perturbagen.

[3] A measure of the enrichment of those instances in the order list of all instances.

[4] A permutation p-value for that enrichment score.

Supplementary Table S2. Association of top-listed drugs from the cmap analysis of db/db mouse liver with diabetes-related diseases or conditions

Drugs	Pharmacology	Molecular mechanisms	References*
Monensin	Carboxylic polyether ionophore	Regulation of the secretion of leptin hormone involved in glucose and insulin metabolism.	[1, 2]
Thapsigargin	Inducer of endoplasmic reticulum stress	Regulation of metabolism in adipocytes and glucose homeostasis in a mouse model of type 2 diabetes.	[3, 4]
Biperiden	Selective muscarinic M1 antagonist	Regulation of memory impairment and control of glucagon-like peptide secretion and insulin release	[5-9]
Adiphenine	Local anesthetic	Inhibition of the activity of the acetylcholine receptor and regulation of glucose metabolism and diabetes	[8-10]
Naringenin	Flavonoid with a various biological activities	Antidiabetic, antiobesity, and immune-modulatory effects<br>Antioxidant and cholinesterase inhibitor against type 2 diabetes-induced memory dysfunction	[11, 12]
Timolol<br>Nadolol	Beta-blocker used in the treatment of cardiovascular diseases	Regulation of hyperglycemia-induced kidney or heart damage in diabetic patients through its antioxidant activities and/or by controlling calcium release	[13-15]

* References

1. Levy, J. R. & Stevens, W. The effects of insulin, glucose, and pyruvate on the kinetics of leptin secretion. Endocrinology 142, 3558-3562 (2001).
2. Benzi, L. et al. Inhibition of endosomal acidification in normal cells mimics the derangements of cellular insulin and insulin-receptor metabolism observed in non-insulin-dependent diabetes mellitus. Metabolism 46, 1259-1265 (1997).
3. Burrill, J. S. et al. Inflammation and ER stress regulate branched-chain amino acid uptake and metabolism in adipocytes. Mol. Endocrinol. 29, 411-420 (2015).
4. Ozcan, U. et al. Chemical chaperones reduce ER stress and restore glucose homeostasis in a mouse model of type 2 diabetes. Science 313, 1137-1140 (2006).
5. Szczodry, O., van der Staay, F. J. & Arndt, S. S. Modelling Alzheimer-like cognitive deficits in rats using biperiden as putative cognition impairer. Behav. Brain Res. 274, 307-311 (2014).
6. Anini, Y. & Brubaker, P. L. Muscarinic receptors control glucagon-like peptide 1 secretion by human endocrine L cells. Endocrinology 144, 3244-3250 (2003).
7. Miguel, J. C., Abdel-Wahab, Y. H., Mathias, P. C. & Flatt, P. R. Muscarinic receptor subtypes mediate

stimulatory and paradoxical inhibitory effects on an insulin-secreting beta cell line.. Biochim. Biophys. Acta. 1569, 45-50 (2002).

8. Gautam, D. et al. Role of the M3 muscarinic acetylcholine receptor in beta-cell function and glucose homeostasis. Diabetes Obes. Metab. 9 Suppl 2:158-169 (2007).

9. Marrero, M. B. et al. An alpha7 nicotinic acetylcholine receptor-selective agonist reduces weight gain and metabolic changes in a mouse model of diabetes. J. Pharmacol. Exp. Ther. 332, 173-180 (2010).

10. Spitzmaul, G., Gumilar, F., Dilger, J. P. & Bouzat, C. The local anaesthetics proadifen and adiphenine inhibit nicotinic receptors by different molecular mechanisms. Br. J. Pharmacol. 157, 804-817 (2009).

11. Yoshida, H. et al. Naringenin suppresses macrophage infiltration into adipose tissue in an early phase of high-fat diet-induced obesity. Biochem. Biophys. Res. Commun. 454, 95-101 (2014).

12. Rahigude, A. et al. Participation of antioxidant and cholinergic system in protective effect of naringenin against type-2 diabetes-induced memory dysfunction in rats. Neuroscience 226, 62-72 (2012).

13. Gokturk, H. et al. Long-term treatment with a beta-blocker timolol attenuates renal-damage in diabetic rats via enhancing kidney antioxidant-defense system. Mol. Cell Biochem. 395, 177-186 (2014).

14. Tuncay, E., Okatan, E. N., Vassort, G. & Turan, B. ß-blocker timolol prevents arrhythmogenic Ca^{2+} release and normalizes Ca^{2+} and Zn^{2+} dyshomeostasis in hyperglycemic rat heart. PLoS One 8, e71014; 10.1371/journal.pone.0071014 (2013).

15. Besana, A., Wang, D. W., George, A. L. Jr. & Schwartz, P. J. Nadolol block of Nav1.5 does not explain its efficacy in the long QT syndrome. J. Cardiovasc. Pharmacol. 59, 249-253 (2012).

Supplementary Table S3. Genes associated with each module.

Liver of db/db mice							HepG2 treated with ESB					
**Module**	**Symbol**	Degree	Betweenness Centrality	Closeness Centrality	Clustering Coefficient		**Module**	**Symbol**	Degree	Betweenness Centrality	Closeness Centrality	Clustering Coefficient
0	NFKB1	46	0.417	0.427	0.051		0	FYN	37	0.115	0.391	0.111
0	UBB	44	0.188	0.385	0.085		0	RAC1	36	0.15	0.399	0.117
0	TRAF6	24	0.07	0.38	0.105		0	RHOA	28	0.087	0.37	0.122
0	PSMD4	20	0.064	0.359	0.289		0	PIK3R2	28	0.077	0.361	0.093
0	PSMB6	13	0.003	0.337	0.654		0	PIK3R1	26	0.077	0.368	0.145
0	PSME2	12	0.002	0.334	0.727		0	GNAI1	21	0.061	0.328	0.11
0	PSME1	12	0.002	0.334	0.727		0	CBL	16	0.031	0.337	0.342
0	PSMF1	12	0.002	0.334	0.727		0	CBLB	15	0.027	0.347	0.429
0	RNF146	7	0	0.294	1		0	RHOG	11	0.004	0.313	0.418
0	TNKS2	7	0	0.294	1		0	PIK3R3	10	0.003	0.318	0.333
0	HECW1	7	0	0.294	0.762		0	CBLC	10	0.003	0.326	0.533
0	NFKBIE	7	0	0.322	1		0	PIK3R5	10	0.003	0.318	0.333
0	GMNN	7	0.022	0.3	0.714		0	CTNND1	9	0.004	0.329	0.417
0	RNF43	3	0	0.279	0.667		0	BLK	8	0.001	0.304	0.607
0	MTPN	2	0	0.305	1		0	RHOB	8	0.004	0.295	0.286
0	PELI2	2	0	0.296	1		0	RHOC	8	0.004	0.295	0.286
0	RSPO3	2	0	0.279	1		0	RND1	7	0.001	0.301	0.429
0	TAX1BP1	2	0	0.296	1		0	DAPP1	7	0	0.308	0.714
0	UBQLN2	2	0	0.265	1		0	PIK3AP1	7	0	0.282	0.571
0	UBQLN1	2	0	0.265	1		0	ARHGEF1	6	0	0.289	0.667
0	ERC1	2	0	0.305	1		0	FGF16	5	0.004	0.307	0.3
0	TLR5	2	0	0.305	1		0	EFNA4	4	0	0.3	1
0	PELI3	2	0	0.296	1		0	EPHA8	4	0	0.3	1
0	PELI1	2	0	0.296	1		0	RTKN	4	0	0.272	0.5
0	LTA	2	0	0.3	1		0	CIT	4	0.002	0.282	0
0	LTB	2	0	0.3	1		0	PI4K2B	4	0	0.274	0
0	TRIM37	1	0	0.3	0		0	ELMO1	3	0	0.297	0.667
0	CSRP1	1	0	0.3	0		0	NOX1	3	0	0.286	1
0	KLK8	1	0	0.278	0		0	CGN	3	0	0.271	1
0	BMF	1	0	0.3	0		0	MYH1	3	0	0.271	1
0	FBXL3	1	0	0.278	0		0	MYH7B	3	0	0.271	1
0	UBQLN4	1	0	0.265	0		0	NOXO1	3	0	0.286	1
0	UBE2K	1	0	0.265	0		0	NOXA1	3	0	0.286	1
0	NFKBID	1	0	0.3	0		0	SH3GLB2	3	0	0.259	1
0	ANKRD42	1	0	0.3	0		0	SH3GLB1	3	0	0.259	1
0	IRAK3	1	0	0.275	0		0	SLA	2	0	0.282	1
0	IDO2	1	0	0.278	0		0	CNTNAP1	2	0	0.293	1
0	PPP2R4	1	0	0.3	0		0	AXL	2	0	0.274	0
0	BEX1	1	0	0.275	0		0	DEF6	2	0	0.288	1
0	TMBIM6	1	0	0.275	0		0	RHOD	2	0	0.266	0
0	REV3L	1	0	0.3	0		0	FARP2	2	0	0.297	1
0	NLRC3	1	0	0.275	0		0	GALR2	2	0	0.247	1
0	UBA7	1	0	0.278	0		0	BRPF3	2	0	0.293	1
0	CSF1	1	0	0.3	0		0	FEZ2	2	0	0.274	0
0	MGLL	1	0	0.3	0		0	ABLIM1	2	0	0.297	1
0	SLC38A2	1	0	0.278	0		0	FMN2	2	0	0.228	0
0	USP1	1	0	0.278	0		0	GAL	2	0	0.247	1
0	AQP1	1	0	0.278	0		0	PXK	1	0	0.269	0
0	SLC12A3	1	0	0.278	0		0	SPRY1	1	0	0.252	0
0	SLC38A4	1	0	0.278	0		0	RXFP3	1	0	0.247	0
0	GPBP1	1	0	0.3	0		0	SNX12	1	0	0.282	0
0	ASXL2	1	0	0.275	0		0	DSCAM	1	0	0.285	0
0	DENND4A	1	0	0.3	0		0	PLEKHG6	1	0	0.27	0
0	TIFA	1	0	0.275	0		0	TULP4	1	0	0.282	0
0	MAOA	1	0	0.278	0		0	DOCK11	1	0	0.285	0
0	SLC11A1	1	0	0.3	0		0	LILRB3	1	0	0.282	0
0	MAOB	1	0	0.278	0		0	FARP1	1	0	0.27	0
0	HSD11B2	1	0	0.3	0		0	RHOT2	1	0	0.265	0
0	OTUD7B	1	0	0.275	0		0	RHOT1	1	0	0.265	0

**Liver of db/db mice**							**HepG2 treated with ESB**					
**Module**	**Symbol**	Degree	Betweenness Centrality	Closeness Centrality	Clustering Coefficient		**Module**	**Symbol**	Degree	Betweenness Centrality	Closeness Centrality	Clustering Coefficient
0	CMPK2	1	0	0.3	0		0	TAS2R50	1	0	0.247	0
0	PIGR	1	0	0.3	0		0	SH2D1A	1	0	0.282	0
1	IL6ST	30	0.044	0.334	0.29		0	CDKL5	1	0	0.282	0
1	LIFR	26	0.033	0.332	0.228		0	GPR143	1	0	0.247	0
1	SOCS3	25	0.056	0.358	0.39		0	ARHGEF10	1	0	0.27	0
1	CNTFR	25	0.148	0.307	0.213		0	RGS11	1	0	0.247	0
1	IL13RA1	24	0.004	0.298	0.178		0	PTGIR	1	0	0.247	0
1	PRLR	24	0.009	0.288	0.087		0	P2RY14	1	0	0.247	0
1	OSMR	23	0.002	0.293	0.162		0	PIR	1	0	0.285	0
1	IL22RA1	23	0.002	0.282	0.162		0	SPATA13	1	0	0.285	0
1	SOCS1	23	0.048	0.344	0.443		0	RHOF	1	0	0.265	0
1	IL10RB	23	0.002	0.282	0.162		0	RHOH	1	0	0.265	0
1	IL22RA2	22	0.002	0.281	0.082		0	RHOU	1	0	0.265	0
1	IL21R	22	0.002	0.281	0.082		0	RHOV	1	0	0.265	0
1	IL15RA	22	0.002	0.281	0.082		0	RHOBTB2	1	0	0.265	0
1	IFNA5	16	0	0.298	0.592		0	UNC5C	1	0	0.282	0
1	IFNA1	16	0	0.298	0.592		0	ARHGEF10L	1	0	0.27	0
1	IFNA7	16	0	0.298	0.592		0	CNKSR1	1	0	0.27	0
1	IFNA14	16	0	0.298	0.592		0	P2RY4	1	0	0.247	0
1	SOCS2	15	0.006	0.308	0.333		0	PTGDR	1	0	0.247	0
1	IL4	14	0.019	0.342	0.231		0	DUSP15	1	0	0.282	0
1	EPO	14	0.019	0.342	0.242		0	RAP1GDS1	1	0	0.285	0
1	IL3	13	0.002	0.288	0.141		0	UGCG	1	0	0.269	0
1	IL15	13	0.019	0.341	0.115		0	MAPK8IP1	1	0	0.282	0
1	CTF1	12	0.002	0.285	0.136		0	RP1L1	1	0	0.282	0
1	CSF3	12	0.017	0.335	0.091		0	GPSM2	1	0	0.247	0
1	IL22	11	0	0.263	0.109		1	CTNNB1	55	0.3	0.394	0.072
1	SOCS4	11	0	0.263	0.109		1	CTBP2	20	0.084	0.308	0.021
1	CNTF	11	0	0.263	0.109		1	WNT1	4	0.011	0.288	0.333
1	SOCS7	11	0	0.263	0.109		1	APC2	3	0	0.283	1
1	IL11	11	0	0.263	0.109		1	PPP6C	3	0	0.283	1
1	IL24	11	0	0.263	0.109		1	FRAT2	3	0	0.283	1
1	IL20	11	0	0.263	0.109		1	SOST	2	0	0.224	1
1	OSM	11	0	0.263	0.109		1	PTPRM	2	0	0.283	1
1	IFNK	11	0	0.263	0.109		1	HINT1	2	0.018	0.307	0
1	CISH	6	0.001	0.321	0.733		1	KREMEN2	2	0	0.224	1
2	PPARA	26	0.053	0.26	0.431		1	CDH8	2	0	0.283	1
2	NCOA1	23	0.078	0.317	0.605		1	BARHL2	1	0	0.236	0
2	MED17	19	0	0.246	0.877		1	EVX1	1	0	0.236	0
2	MED12	19	0	0.246	0.877		1	NKX6-1	1	0	0.236	0
2	NCOA3	19	0.059	0.316	0.865		1	VEZT	1	0	0.283	0
2	MED13	19	0	0.246	0.877		1	OTP	1	0	0.236	0
2	MED22	18	0	0.246	0.961		1	WNT2	1	0	0.283	0
2	MED25	18	0	0.246	0.961		1	IGFBP2	1	0	0.283	0
2	MED9	18	0	0.246	0.961		1	TBX1	1	0	0.236	0
2	MED14	18	0	0.246	0.961		1	SOX13	1	0	0.236	0
2	MED15	18	0	0.246	0.961		1	SOX14	1	0	0.236	0
2	MED29	18	0	0.246	0.961		1	LGALS9	1	0	0.283	0
2	MED24	18	0	0.246	0.961		1	L3MBTL3	1	0	0.283	0
2	MED27	18	0	0.246	0.961		1	GBX2	1	0	0.236	0
2	CDK19	18	0	0.246	0.961		1	ACP1	1	0	0.283	0
2	TGS1	16	0	0.246	1		1	FHIT	1	0	0.283	0
2	NCOA6	16	0	0.246	1		1	WNT10B	1	0	0.283	0
2	CHD9	15	0	0.245	1		1	WNT10A	1	0	0.283	0
2	FABP4	14	0	0.243	1		1	WNT16	1	0	0.283	0
2	TRIP4	5	0.006	0.309	0.7		1	WNT2B	1	0	0.283	0
2	CPT1C	4	0	0.207	0.5		1	OTX1	1	0	0.236	0
2	CPT1A	4	0	0.207	0.5		1	LBX1	1	0	0.236	0
2	ACSBG2	3	0	0.207	0.667		1	FOXJ1	1	0	0.236	0

Liver of db/db mice							HepG2 treated with ESB					
**Module**	**Symbol**	Degree	Betweenness Centrality	Closeness Centrality	Clustering Coefficient		**Module**	**Symbol**	Degree	Betweenness Centrality	Closeness Centrality	Clustering Coefficient
2	ACSBG1	3	0	0.207	0.667		1	WNT9B	1	0	0.283	0
2	ACSL3	3	0	0.207	0.667		1	KLF8	1	0	0.236	0
2	FABP6	2	0	0.241	1		1	IRX5	1	0	0.236	0
2	NR1H3	2	0.019	0.292	0		1	RANBP3	1	0	0.283	0
2	SLC10A2	2	0	0.241	1		1	LHX9	1	0	0.236	0
2	LIPA	1	0	0.206	0		1	SHOX2	1	0	0.236	0
2	CYP7B1	1	0	0.241	0		1	ARX	1	0	0.236	0
2	CPT2	1	0	0.206	0		1	DLG5	1	0	0.283	0
2	ADIPOQ	1	0	0.206	0		2	CUL5	25	0.06	0.27	0.107
3	GRB2	32	0.069	0.354	0.153		2	RELA	23	0.114	0.384	0.202
3	LCK	22	0.1	0.382	0.156		2	TCEB2	20	0.039	0.274	0.168
3	GAB2	17	0.015	0.338	0.294		2	PSMB5	16	0.023	0.321	0.483
3	FRS2	9	0.003	0.317	0.611		2	PSMB8	15	0.027	0.326	0.552
3	SHC3	8	0.001	0.284	0.607		2	PSMC4	14	0.013	0.32	0.637
3	BLK	6	0.003	0.326	0.133		2	PSMF1	14	0.026	0.331	0.626
3	GRAP	6	0	0.265	0.6		2	PSMB2	13	0.011	0.32	0.718
3	FGF22	6	0.002	0.316	0.867		2	PSME2	13	0.011	0.32	0.718
3	FGF9	6	0.002	0.316	0.867		2	REL	10	0.003	0.314	0.822
3	PRKD3	5	0	0.265	0.9		2	NFKBIE	8	0	0.287	1
3	SHC2	4	0	0.283	0.833		2	RFWD2	6	0	0.254	1
3	DOK1	4	0	0.286	0.333		2	PTTG1	6	0	0.254	1
3	RASAL2	4	0	0.265	1		2	KRT8	4	0.004	0.287	0.5
3	NF1	4	0	0.265	1		2	ASB16	3	0.012	0.297	0.333
3	CD3E	3	0	0.277	1		2	WSB2	2	0	0.216	1
3	PDCD1LG2	3	0	0.277	1		2	ASB5	2	0	0.216	1
3	TRPV6	3	0	0.3	0.667		2	ASB9	2	0	0.216	1
3	PDCD1	3	0	0.277	1		2	ASB10	2	0	0.216	1
3	SGMS1	3	0	0.286	1		2	ASB12	2	0	0.216	1
3	TFF3	2	0.001	0.313	0		2	ASB8	2	0	0.216	1
3	STAP1	2	0	0.277	0		2	ASB3	2	0	0.216	1
3	LIME1	1	0	0.277	0		2	ASB17	2	0	0.216	1
3	CD7	1	0	0.277	0		2	ASB13	2	0	0.216	1
3	PTPN22	1	0	0.277	0		2	ASB2	2	0	0.216	1
3	PDGFD	1	0	0.262	0		2	KLHL15	1	0	0.213	0
3	TFF2	1	0	0.262	0		2	PCNP	1	0	0.213	0
3	WIPF3	1	0	0.262	0		2	KLF5	1	0	0.278	0
3	WIPF2	1	0	0.262	0		2	TMBIM4	1	0	0.243	0
3	WIPF1	1	0	0.262	0		2	CRIP2	1	0	0.278	0
4	RAC2	28	0.158	0.324	0.005		2	COQ2	1	0	0.243	0
4	SEMA4D	2	0	0.245	1		2	UXT	1	0	0.278	0
4	ARHGAP9	2	0.008	0.3	0		2	RAB40B	1	0	0.213	0
4	ARHGAP5	2	0.006	0.246	0		2	NFKBIZ	1	0	0.278	0
4	RND1	2	0	0.245	1		2	SPSB1	1	0	0.213	0
4	SYDE2	1	0	0.245	0		2	SPSB3	1	0	0.213	0
4	ARHGAP11A	1	0	0.245	0		2	IL18R1	1	0	0.278	0
4	SYDE1	1	0	0.245	0		2	DCUN1D1	1	0	0.213	0
4	FAM13A	1	0	0.245	0		2	BTBD1	1	0	0.213	0
4	ICMT	1	0	0.245	0		2	WSB1	1	0	0.215	0
4	THY1	1	0	0.197	0		3	NRF1	15	0.105	0.299	0.01
4	TAGAP	1	0	0.245	0		3	SETDB1	14	0.065	0.274	0
4	OPHN1	1	0	0.245	0		3	YY1	13	0.061	0.251	0.013
4	FAM13B	1	0	0.245	0		3	SAFB2	2	0	0.239	1
4	MYO9B	1	0	0.245	0		3	ZNF614	2	0.002	0.231	0
4	NET1	1	0	0.245	0		3	ZNF613	2	0.002	0.231	0
4	DIRAS1	1	0	0.245	0		3	DEDD2	1	0	0.23	0
4	ARHGEF16	1	0	0.245	0		3	ALKBH5	1	0	0.23	0
4	FGD1	1	0	0.245	0		3	PITPNB	1	0	0.23	0
4	ARHGEF3	1	0	0.245	0		3	GPBP1L1	1	0	0.23	0
4	ARHGAP23	1	0	0.245	0		3	ERH	1	0	0.201	0

Liver of db/db mice						HepG2 treated with ESB					
**Module**	**Symbol**	Degree	Betweenness Centrality	Closeness Centrality	Clustering Coefficient	**Module**	**Symbol**	Degree	Betweenness Centrality	Closeness Centrality	Clustering Coefficient
4	MYO9A	1	0	0.245	0	3	ALKBH8	1	0	0.201	0
4	GEM	1	0	0.245	0	3	NDRG3	1	0	0.201	0
4	ARHGEF17	1	0	0.245	0	3	ZFP64	1	0	0.215	0
4	CHN2	1	0	0.245	0	3	ZNF441	1	0	0.215	0
4	DEPDC7	1	0	0.245	0	3	ZNF524	1	0	0.201	0
4	ARHGAP29	1	0	0.245	0	3	ZNF416	1	0	0.215	0
4	ARHGAP19	1	0	0.245	0	3	ZNF436	1	0	0.215	0
5	SUZ12	24	0.14	0.202	0.011	3	ZNF433	1	0	0.215	0
5	NEUROG3	3	0	0.168	0.667	3	ZNF669	1	0	0.215	0
5	NKX2-2	3	0	0.168	0.667	3	SETDB2	1	0	0.215	0
5	ONECUT1	2	0	0.168	1	3	ETFDH	1	0	0.23	0
5	NKX6-1	2	0	0.168	1	3	FAM120B	1	0	0.23	0
5	CRLF1	2	0.14	0.244	0	3	ZNF32	1	0	0.215	0
5	SOX14	1	0	0.168	0	3	ZNF165	1	0	0.215	0
5	PTF1A	1	0	0.168	0	3	FBXO31	1	0	0.201	0
5	ONECUT2	1	0	0.168	0	3	ZNF576	1	0	0.23	0
5	PRDM12	1	0	0.168	0	3	ZNF134	1	0	0.215	0
5	HMX2	1	0	0.168	0	3	LYPLA1	1	0	0.23	0
5	NKX3-2	1	0	0.168	0	3	ZNF432	1	0	0.201	0
5	VSX2	1	0	0.168	0	3	ZNF689	1	0	0.201	0
5	FOXC2	1	0	0.168	0	3	AMACR	1	0	0.23	0
5	OSR2	1	0	0.168	0	3	ABHD5	1	0	0.23	0
5	HOXA6	1	0	0.168	0	3	ZNF551	1	0	0.215	0
5	BARHL1	1	0	0.168	0	3	ZBTB11	1	0	0.201	0
5	GSX1	1	0	0.168	0	4	PAFAH1B1	27	0.085	0.329	0.399
5	ZIC1	1	0	0.168	0	4	TUBB	24	0.073	0.292	0.453
5	GBX2	1	0	0.168	0	4	NDE1	22	0.013	0.291	0.602
5	FOXL2	1	0	0.168	0	4	CEP164	19	0.027	0.267	0.702
5	VAX1	1	0	0.168	0	4	PLK4	17	0.002	0.26	0.86
5	RFX4	1	0	0.168	0	4	CEP72	17	0.005	0.261	0.86
5	HMX1	1	0	0.168	0	4	CEP78	16	0	0.26	0.975
5	LBX2	1	0	0.168	0	4	NEDD1	16	0	0.26	0.975
6	SMC3	20	0.12	0.297	0.395	4	CDK5RAP2	16	0	0.26	0.975
6	STAG2	15	0	0.231	0.714	4	CEP57	16	0	0.26	0.975
6	STAG1	15	0	0.231	0.714	4	ODF2	16	0	0.26	0.975
6	PDS5B	13	0	0.231	0.897	4	PCNT	16	0	0.26	0.975
6	WAPAL	13	0	0.231	0.897	4	SSNA1	16	0	0.26	0.975
6	ZW10	13	0.006	0.231	0.846	4	TUBGCP5	15	0	0.26	1
6	CENPO	12	0	0.231	1	4	TUBGCP2	15	0	0.26	1
6	NDC80	12	0	0.231	1	4	TUBGCP3	15	0	0.26	1
6	NUDC	12	0	0.231	1	4	XPO1	15	0.079	0.338	0.238
6	TAOK1	12	0	0.231	1	4	TMEM67	13	0	0.259	1
6	CENPI	12	0	0.231	1	4	PMF1	8	0.004	0.305	0.786
6	DSN1	12	0	0.231	1	4	RANGAP1	8	0.018	0.283	0.75
6	AHCTF1	12	0	0.231	1	4	TAOK1	7	0	0.276	1
6	ESCO1	5	0	0.23	1	4	CENPL	7	0	0.276	1
6	SYCP3	4	0	0.229	1	4	SGOL2	7	0	0.276	1
6	ACD	4	0	0.229	1	4	MX1	2	0.005	0.282	0
6	P2RX6	1	0	0.229	0	4	DCX	2	0.003	0.3	0
6	FEZ2	1	0	0.229	0	4	KIF1C	1	0	0.226	0
6	GPRC5A	1	0	0.229	0	4	XPO7	1	0	0.253	0
6	RINT1	1	0	0.188	0	4	MAP1S	1	0	0.248	0
6	CCDC124	1	0	0.229	0	4	DCPS	1	0	0.253	0
7	MAPK1	36	0.266	0.4	0.079	4	TNFSF13	1	0	0.253	0
7	MAPK3	28	0.044	0.368	0.124	4	PLA2G7	1	0	0.248	0
7	VRK3	3	0	0.287	1	4	OTUD7B	1	0	0.253	0
7	DUSP3	3	0	0.287	1	5	TRAF2	24	0.167	0.358	0.109
7	IER3	2	0	0.326	1	5	UBE2D2	21	0.088	0.293	0.029
7	PLA2G5	2	0	0.287	1	5	TRAF1	10	0.005	0.317	0.222

Liver of db/db mice							HepG2 treated with ESB					
**Module**	**Symbol**	Degree	Betweenness Centrality	Closeness Centrality	Clustering Coefficient		**Module**	**Symbol**	Degree	Betweenness Centrality	Closeness Centrality	Clustering Coefficient
7	PLA2G6	2	0	0.287	1		5	UBE2D4	3	0.001	0.27	0.667
7	PLA2G3	2	0	0.287	1		5	RFFL	2	0	0.27	1
7	PDYN	2	0	0.287	1		5	TRIM23	2	0	0.227	1
7	PLA2G16	2	0	0.287	1		5	ZMAT3	2	0	0.264	1
7	PLA1A	2	0	0.287	1		5	TNFRSF18	2	0	0.264	1
7	PLA2G2F	2	0	0.287	1		5	MKRN3	2	0	0.227	1
7	PLA2G2E	2	0	0.287	1		5	TNFRSF19	2	0	0.264	1
7	PLA2G2D	2	0	0.287	1		5	RNF185	2	0.005	0.227	0
7	PTPRR	2	0	0.287	1		5	TNFSF9	2	0	0.264	1
7	EIF4EBP2	2	0	0.287	1		5	RNF126	2	0	0.227	1
7	CROCC	2	0	0.287	1		5	SMPD3	1	0	0.264	0
7	RPS6KA4	2	0	0.287	1		5	UBOX5	1	0	0.227	0
7	GABRR1	1	0	0.286	0		5	RIPK4	1	0	0.264	0
7	CAPNS1	1	0	0.286	0		5	RNF25	1	0	0.227	0
7	CMTM3	1	0	0.286	0		5	PJA2	1	0	0.227	0
8	VCP	20	0.095	0.318	0.068		5	TRIM26	1	0	0.227	0
8	DERL2	5	0.002	0.294	0.7		5	ZFAND6	1	0	0.264	0
8	NPLOC4	5	0	0.242	0.4		5	EDARADD	1	0	0.264	0
8	ERLEC1	4	0	0.294	1		5	RNF138	1	0	0.227	0
8	SEL1L	4	0	0.294	1		5	PDZRN3	1	0	0.227	0
8	SVIP	3	0	0.242	0.667		5	UBE2E3	1	0	0.185	0
8	YOD1	3	0	0.242	0.667		5	RNF125	1	0	0.227	0
8	UBE2J1	3	0	0.242	0.667		5	MID2	1	0	0.227	0
8	UBXN8	2	0	0.242	1		5	DZIP3	1	0	0.227	0
8	UBXN6	2	0	0.242	1		5	TNFSF4	1	0	0.264	0
8	UFD1L	2	0	0.242	1		5	RNF151	1	0	0.227	0
8	RNF19A	1	0	0.242	0		5	MYLIP	1	0	0.227	0
8	CDKN2AIP	1	0	0.242	0		6	MAPK1	25	0.168	0.357	0.1
8	VCPIP1	1	0	0.242	0		6	MAPK3	20	0.025	0.333	0.137
8	UBXN2B	1	0	0.242	0		6	STAT1	17	0.084	0.349	0.029
8	UBXN2A	1	0	0.242	0		6	S100A12	3	0	0.3	1
8	UBXN1	1	0	0.242	0		6	PEA15	2	0	0.264	1
8	MDN1	1	0	0.242	0		6	PGF	2	0	0.291	0
8	RNF19B	1	0	0.242	0		6	PLA2G5	2	0	0.264	1
9	RPS3	13	0.049	0.307	0.538		6	PLA2G6	2	0	0.264	1
9	RPL7	11	0.007	0.27	0.782		6	PLA1A	2	0	0.264	1
9	RPL9	11	0.007	0.27	0.782		6	PTPN7	2	0	0.264	1
9	RPS21	10	0	0.238	0.933		6	DUSP5	2	0	0.264	1
9	RPL12	10	0	0.238	0.933		6	PLA2G2F	2	0	0.264	1
9	RPL35	10	0	0.238	0.933		6	HERC6	2	0.005	0.26	0
9	RPL8	10	0	0.238	0.933		6	CROCC	2	0	0.264	1
9	RPS15	9	0	0.238	1		6	DUSP7	2	0	0.264	1
9	RPL26L1	9	0	0.238	1		6	VRK3	2	0	0.264	1
9	RPL3	9	0	0.238	1		6	DUSP16	2	0	0.264	1
9	RPL10L	7	0	0.238	1		6	BST2	1	0	0.259	0
9	SLC20A2	1	0	0.235	0		6	HERC3	1	0	0.206	0
9	TXNL4B	1	0	0.235	0		6	PNPT1	1	0	0.259	0
10	RBX1	22	0.08	0.354	0.121		6	POR	1	0	0.259	0
10	KLHL9	3	0	0.262	0.667		6	NNMT	1	0	0.259	0
10	KLHL21	2	0	0.262	1		6	CMTM3	1	0	0.264	0
10	KLHL13	2	0	0.262	1		6	LY6E	1	0	0.259	0
10	ASB16	1	0	0.262	0		6	OAS1	1	0	0.259	0
10	ZC3HC1	1	0	0.262	0		7	PRKAB2	22	0.084	0.286	0.333
10	FBXW2	1	0	0.262	0		7	PRKAA1	20	0.012	0.241	0.405
10	ASB13	1	0	0.262	0		7	PRKAB1	19	0.018	0.257	0.45
10	FBXL12	1	0	0.262	0		7	PRKAA2	18	0.002	0.241	0.503
10	ENC1	1	0	0.262	0		7	PRKAG2	18	0.002	0.241	0.503
10	FBXO33	1	0	0.262	0		7	PRKAG1	18	0.002	0.241	0.503
10	ASB18	1	0	0.262	0		7	ADIPOR1	8	0	0.23	0.964

Liver of db/db mice

**Module**	**Symbol**	Degree	Betweenness Centrality	Closeness Centrality	Clustering Coefficient
10	CUL4B	1	0	0.262	0
11	RING1	10	0.013	0.214	0.333
11	RNF2	10	0.013	0.214	0.333
11	BMI1	6	0.001	0.214	0.733
11	CBX7	6	0.045	0.261	0.533
11	SCMH1	5	0.017	0.237	0.6
11	PCGF6	4	0	0.209	1
11	PHC1	4	0	0.194	1
11	PCGF5	3	0	0.208	1
11	TCEA3	2	0	0.177	1
11	EPC2	2	0	0.177	1
11	COMMD3	1	0	0.177	0
11	SCML2	1	0	0.177	0
12	TPR	12	0.087	0.344	0.485
12	NUPL1	10	0.011	0.294	0.667
12	NUP93	9	0.006	0.259	0.778
12	SNUPN	8	0	0.259	1
12	NUP210	8	0	0.259	1
12	NUP50	8	0	0.259	1
12	NUP188	8	0	0.259	1
12	NUP88	8	0	0.259	1
12	POM121	8	0	0.259	1
12	ANO4	1	0	0.206	0

HepG2 treated with ESB

**Module**	**Symbol**	Degree	Betweenness Centrality	Closeness Centrality	Clustering Coefficient
7	PFKFB4	7	0.019	0.261	0.714
7	CAMKK1	7	0	0.23	1
7	CAMKK2	7	0	0.23	1
7	G6PC	6	0	0.23	1
7	PFKFB2	6	0	0.23	1
7	G6PC3	6	0	0.23	1
7	AGRP	6	0	0.23	1
7	EEF2K	6	0	0.23	1
7	G6PC2	6	0	0.23	1
7	PFKFB3	6	0	0.23	1
7	MLYCD	6	0	0.23	1
7	PFKFB1	6	0	0.23	1
7	MAP3K15	1	0	0.223	0
7	GRN	1	0	0.223	0
7	SLC25A13	1	0	0.195	0
7	DAO	1	0	0.223	0
7	PTPLAD1	1	0	0.195	0
8	RPA2	20	0.077	0.269	0.211
8	RPA3	13	0	0.213	0.295
8	RAD17	8	0.006	0.256	0.536
8	TOPBP1	7	0.004	0.256	0.667
8	RPA4	7	0	0.213	0.714
8	FANCG	7	0	0.213	0.619
8	FANCC	6	0	0.212	0.8
8	RMI1	6	0	0.212	0.6
8	MCM10	6	0.003	0.23	0.667
8	FANCD2	5	0	0.212	1
8	DBF4	5	0	0.212	1
8	PRIM1	5	0	0.212	1
8	ERCC1	4	0	0.212	0.667
8	TOP3B	4	0	0.212	1
8	SMARCAL1	4	0.056	0.3	0.5
8	XPC	3	0	0.212	1
8	MND1	3	0	0.212	1
8	PSMC3IP	3	0	0.212	1
8	HSBP1	2	0	0.212	1
9	CDH3	14	0	0.286	0.462
9	CELSR3	10	0.002	0.31	0.822
9	CDH15	10	0	0.285	0.844
9	PCDH11X	9	0	0.285	1
9	PCDHB12	9	0	0.285	1
9	PCDHB11	9	0	0.285	1
9	PCDHB10	9	0	0.285	1
9	PCDHB4	9	0	0.285	1
9	PCDHGA2	9	0	0.285	1
9	MMP21	2	0	0.283	1
9	CBLL1	2	0	0.283	1
9	PKP4	2	0	0.283	1
9	WNT3	2	0	0.283	1
10	ARF1	13	0.053	0.312	0.231
10	ARF3	7	0.001	0.252	0.667
10	ARF5	7	0.001	0.252	0.667
10	RAB25	6	0	0.252	0.8
10	TUBB8	5	0.007	0.242	0.6
10	RAB2B	4	0	0.239	1
10	ARFGAP3	4	0.002	0.294	0.5
10	COPA	3	0	0.24	0.667
10	CADPS2	3	0	0.238	1
10	ARCN1	2	0	0.238	1

Liver of db/db mice							HepG2 treated with ESB					
**Module**	**Symbol**	Degree	Betweenness Centrality	Closeness Centrality	Clustering Coefficient		**Module**	**Symbol**	Degree	Betweenness Centrality	Closeness Centrality	Clustering Coefficient
							10	GRASP	1	0	0.238	0
							11	DYNLL2	11	0.052	0.229	0.655
							11	IFT81	10	0.001	0.187	0.978
							11	IFT80	10	0.001	0.187	0.978
							11	WDR19	10	0.001	0.187	0.978
							11	WDR60	10	0.001	0.187	0.978
							11	DYNC2LI1	10	0.001	0.187	0.978
							11	TTC26	10	0.001	0.187	0.978
							11	KIF3C	10	0.001	0.187	0.978
							11	WDR35	10	0.001	0.187	0.978
							11	TTC30B	10	0.001	0.187	0.978
							11	DYNLRB1	9	0	0.158	1
							12	PCBP2	10	0.048	0.3	0.467
							12	PCBP1	9	0.007	0.233	0.639
							12	PTBP1	8	0.002	0.232	0.821
							12	SNRPA	8	0.002	0.232	0.821
							12	PCF11	7	0	0.232	1
							12	PLRG1	7	0	0.232	1
							12	EFTUD2	7	0	0.232	1
							12	SNRPA1	7	0	0.232	1
							12	RALY	3	0	0.189	1
							12	RBM11	1	0	0.189	0

Supplementary Table S4. The biological functions associated with each module.

<List of GO terms>

	**Liver of db/db mice**				**HepG2 treated with ESB**	
**Module**	**GO**	**FDR**		**Module**	**GO**	**FDR**
0	Toll signaling pathway	1.43E-04		0	Rho protein signal transduction	1.43E-04
0	Positive regulation of NF-kB transcription factor activity	1.67E-04		0	Regulation of phosphatidylinositol 3-kinase activity	1.67E-04
0	Toll-like receptor signaling pathway	2.00E-04		0	Positive regulation of Rho GTPase activity	2.00E-04
0	Toll-like receptor 2 signaling pathway	2.31E-04		0	Regulation of respiratory burst	2.22E-04
0	Negative regulation of NF-kB transcription factor activity	2.50E-04		0	Regulation of hydrogen peroxide metabolic process	2.22E-04
0	Negative regulation of type I interferon production	2.50E-04		0	Axon guidance	2.50E-04
0	Toll-like receptor 9 signaling pathway	2.73E-04		0	GTP catabolic process	3.33E-04
0	Protein polyubiquitination	3.00E-04		0	Regulation of small GTPase mediated signal transduction	5.00E-04
0	Toll-like receptor 10 signaling pathway	3.33E-04		0	Rac protein signal transduction	5.00E-04
0	Toll-like receptor 5 signaling pathway	3.33E-04		0	Neuron remodeling	7.27E-04
0	Toll-like receptor TLR1:TLR2 signaling pathway	3.33E-04		0	Small GTPase mediated signal transduction	1.00E-03
0	Toll-like receptor TLR6:TLR2 signaling pathway	3.33E-04		0	Platelet activation	1.25E-03
0	MyD88-dependent toll-like receptor signaling pathway	1.00E-03		0	Dendrite morphogenesis	2.46E-03
0	Toll-like receptor 4 signaling pathway	1.40E-03		0	Positive regulation of Rac GTPase activity	2.67E-03
0	Response to lipopolysaccharide	1.44E-03		0	Lamellipodium assembly	2.79E-03
0	i-kB kinase/NF-kB cascade	1.50E-03		1	Positive regulation of mesenchymal cell proliferation	2.00E-04
0	DNA damage response, signal transduction by p53 class mediator resulting in cell cycle arrest	4.82E-03		1	Cell fate commitment	2.50E-04
0	Positive regulation of osteoclast differentiation	7.74E-03		1	Lung induction	3.33E-04
0	Negative regulation of ubiquitin-protein ligase activity involved in mitotic cell cycle	8.17E-03		1	Canonical Wnt receptor signaling pathway	3.33E-04
0	Positive regulation of ubiquitin-protein ligase activity involved in mitotic cell cycle	9.19E-03		1	Neuron differentiation	5.00E-04
0	Negative regulation of apoptotic process	9.55E-03		1	Wnt receptor signaling pathway	1.00E-03
1	Response to cytokine stimulus	3.85E-05		1	Inner ear morphogenesis	1.57E-03
1	Positive regulation of acute inflammatory response	4.00E-05		1	Cellular response to retinoic acid	3.75E-03
1	Defense response to virus	4.17E-05		1	Regulation of transcription from RNA polymerase II promoter involved in ventral spinal cord interneuron specification	7.67E-03
1	JAK-STAT cascade	4.35E-05		2	Anaphase-promoting complex-dependent proteasomal ubiquitin-dependent protein catabolic process	2.50E-04
1	Negative regulation of protein kinase activity	4.55E-05		2	DNA damage response, signal transduction by p53 class mediator resulting in cell cycle arrest	3.33E-04
1	Negative regulation of insulin receptor signaling pathway	4.76E-05		2	Intracellular signal transduction	5.00E-04
1	Humoral immune response	5.00E-05		2	Regulation of cellular amino acid metabolic process	6.00E-04
1	Leukemia inhibitory factor signaling pathway	5.26E-05		2	Protein polyubiquitination	8.33E-04
1	Regulation of growth	5.56E-05		2	Protein ubiquitination	1.00E-03
1	Adaptive immune response	5.88E-05		2	Negative regulation of ubiquitin-protein ligase activity involved in mitotic cell cycle	2.57E-03
1	Response to exogenous dsRNA	6.25E-05		2	Regulation of ubiquitin-protein ligase activity involved in mitotic cell cycle	3.73E-03
1	Type I interferon-mediated signaling pathway	6.67E-05		2	Antigen processing and presentation of exogenous peptide antigen via MHC class I, TAP-dependent	4.10E-03
1	B cell proliferation	7.14E-05		2	G1/S transition of mitotic cell cycle	4.11E-03
1	Positive regulation of tyrosine phosphorylation of Stat3 protein	7.69E-05		2	Positive regulation of ubiquitin-protein ligase activity involved in mitotic cell cycle	4.50E-03
1	JAK-STAT cascade involved in growth hormone signaling pathway	8.33E-05		2	Antigen processing and presentation of exogenous peptide antigen via MHC class I	5.17E-03
1	Positive regulation of peptidyl-serine phosphorylation of STAT protein	9.09E-05		3	Regulation of transcription, DNA-dependent	5.00E-04
1	T cell activation involved in immune response	1.00E-04		3	Transcription, DNA-dependent	1.00E-03
1	Regulation of MHC class I biosynthetic process	1.00E-04		4	Microtubule cytoskeleton organization	3.33E-04
1	Natural killer cell activation involved in immune response	1.00E-04		4	G2/M transition of mitotic cell cycle	5.00E-04

1	Ciliary neurotrophic factor-mediated signaling pathway	1.43E-04		4	Mitotic cell cycle	5.00E-04
1	Oncostatin-M-mediated signaling pathway	1.43E-04		4	Microtubule nucleation	7.50E-04
1	Positive regulation of tyrosine phosphorylation of Stat5 protein	1.85E-04		4	Establishment of mitotic spindle orientation	8.00E-04
1	B cell differentiation	2.00E-04		5	Positive regulation of NF-kB transcription factor activity	3.33E-04
1	Regulation of type I interferon-mediated signaling pathway	2.50E-04		5	Protein K48-linked ubiquitination	5.00E-04
1	Positive regulation of cell proliferation	3.33E-04		5	Protein ubiquitination	1.00E-03
1	Immune response	4.29E-04		5	Tumor necrosis factor-mediated signaling pathway	7.75E-03
1	Negative regulation of JAK-STAT cascade	5.00E-04		5	Protein K63-linked ubiquitination	8.20E-03
1	Cytokine-mediated signaling pathway	1.00E-03		6	Inactivation of MAPK activity	5.33E-03
1	Cell-cell signaling	1.07E-03		6	Phosphatidylcholine acyl-chain remodeling	7.00E-03
1	Positive regulation of peptidyl-tyrosine phosphorylation	1.13E-03		6	Peptidyl-tyrosine dephosphorylation	7.25E-03
1	Protein ubiquitination	3.61E-03		6	Phosphatidylethanolamine acyl-chain remodeling	1.00E-02
1	Positive regulation of natural killer cell differentiation	6.97E-03		7	Membrane organization	6.25E-05
1	Fat cell differentiation	7.30E-03		7	Cell cycle arrest	6.67E-05
1	Positive regulation of T cell proliferation	8.29E-03		7	Glucose metabolic process	7.14E-05
2	Positive regulation of transcription, DNA-dependent	1.25E-04		7	Carnitine shuttle	7.69E-05
2	Fatty acid beta-oxidation	1.43E-04		7	Glycolysis	8.33E-05
2	Cellular lipid metabolic process	1.67E-04		7	Regulation of fatty acid biosynthetic process	9.09E-05
2	Stem cell maintenance	2.00E-04		7	Insulin receptor signaling pathway	1.00E-04
2	Long-chain fatty acid metabolic process	2.50E-04		7	Protein phosphorylation	1.11E-04
2	Transcription initiation from RNA polymerase II promoter	3.33E-04		7	Carbohydrate phosphorylation	1.25E-04
2	Regulation of transcription from RNA polymerase II promoter	3.33E-04		7	Small molecule metabolic process	1.43E-04
2	Intracellular steroid hormone receptor signaling pathway	5.00E-04		7	Carbohydrate metabolic process	1.67E-04
2	Androgen receptor signaling pathway	1.00E-03		7	Gluconeogenesis	2.00E-04
2	Labyrinthine layer morphogenesis	3.60E-03		7	Dephosphorylation	2.50E-04
2	Bile acid metabolic process	6.00E-03		7	Fructose metabolic process	3.33E-04
2	Intracellular receptor mediated signaling pathway	7.67E-03		7	Fructose 2,6-bisphosphate metabolic process	5.00E-04
2	Gene expression	8.50E-03		7	Glucose-6-phosphate transport	9.41E-04
2	Carnitine metabolic process	8.54E-03		7	Energy reserve metabolic process	9.44E-04
3	Insulin receptor signaling pathway	5.00E-04		7	Fatty acid biosynthetic process	1.00E-03
3	Transmembrane receptor protein tyrosine kinase signaling pathway	1.00E-03		7	Positive regulation of glucokinase activity	2.21E-03
3	Ras protein signal transduction	6.33E-03		7	Hexose transport	2.60E-03
4	Apoptotic signaling pathway	2.00E-04		7	Regulation of glycolysis	2.81E-03
4	Positive regulation of GTPase activity	2.50E-04		7	Cellular lipid metabolic process	3.36E-03
4	Positive regulation of Rho GTPase activity	3.33E-04		7	Glucose 6-phosphate metabolic process	3.48E-03
4	Small GTPase mediated signal transduction	5.00E-04		7	Glucose transport	5.33E-03
4	Regulation of small GTPase mediated signal transduction	5.00E-04		8	G1/S transition of mitotic cell cycle	2.50E-04
5	Negative regulation of transcription from RNA polymerase II promoter	2.50E-04		8	Nucleotide-excision repair	3.33E-04
5	Positive regulation of transcription from RNA polymerase II promoter	3.33E-04		8	Nucleotide-excision repair, DNA damage removal	4.00E-04
5	Endocrine pancreas development	5.00E-04		8	DNA replication	5.00E-04
5	Transcription, DNA-dependent	1.00E-03		8	DNA repair	1.00E-03
5	Pancreas development	2.20E-03		8	DNA recombinase assembly	2.17E-03
6	Mitosis	3.33E-04		9	Homophilic cell adhesion	1.00E-03
6	Cell division	5.00E-04		9	Calcium-dependent cell-cell adhesion	1.00E-03
6	Mitotic cell cycle	1.00E-03		9	Synapse assembly	3.67E-03
7	Phosphatidic acid biosynthetic process	1.11E-04		9	Cell adhesion	7.00E-03
7	Phospholipid metabolic process	1.25E-04		10	Retrograde vesicle-mediated transport, Golgi to ER	2.50E-04
7	Phosphatidylinositol acyl-chain remodeling	1.43E-04		10	GTP catabolic process	3.33E-04
7	Phosphatidylserine acyl-chain remodeling	1.43E-04		10	Intracellular protein transport	5.00E-04
7	Phosphatidylglycerol acyl-chain remodeling	1.43E-04		10	COPI coating of Golgi vesicle	1.00E-03
7	Glycerophospholipid biosynthetic process	2.50E-04		11	Intraflagellar transport	5.00E-04
7	Lipid catabolic process	3.33E-04		11	Cilium assembly	1.00E-03
7	Phosphatidylcholine acyl-chain remodeling	5.00E-04		11	Metabolic process	4.80E-03
7	Phosphatidylethanolamine acyl-chain remodeling	1.00E-03		11	Antigen processing and presentation of exogenous peptide antigen via MHC class II	6.00E-03
7	Negative regulation of ERK1 and ERK2 cascade	1.50E-03		11	Intraflagellar retrograde transport	6.33E-03

8	ER-associated protein catabolic process	1.00E-03		12	RNA splicing	5.00E-04
8	nuclear envelope reassembly	4.00E-03		12	gene expression	6.67E-04
8	membrane fusion	4.67E-03		12	nuclear mRNA splicing, via spliceosome	1.00E-03
8	protein K11-linked deubiquitination	6.25E-03				
8	Golgi organization	8.80E-03				
9	gene expression	7.69E-05				
9	cellular protein metabolic process	8.33E-05				
9	viral reproduction	9.09E-05				
9	RNA metabolic process	1.00E-04				
9	mRNA metabolic process	1.11E-04				
9	translational initiation	1.25E-04				
9	nuclear-transcribed mRNA catabolic process, nonsense-mediated decay	1.43E-04				
9	viral infectious cycle	1.67E-04				
9	SRP-dependent cotranslational protein targeting to membrane	2.00E-04				
9	translational elongation	2.50E-04				
9	translational termination	3.33E-04				
9	viral transcription	5.00E-04				
9	translation	1.00E-03				
10	protein ubiquitination	1.00E-03				
11	transcription, DNA-dependent	1.00E-03				
12	protein import into nucleus	7.69E-05				
12	small molecule metabolic process	8.33E-05				
12	protein transport	9.09E-05				
12	viral reproduction	1.00E-04				
12	mitotic cell cycle	1.11E-04				
12	carbohydrate metabolic process	1.25E-04				
12	mRNA transport	1.43E-04				
12	cytokine-mediated signaling pathway	1.67E-04				
12	transmembrane transport	2.00E-04				
12	regulation of glucose transport	2.50E-04				
12	mitotic nuclear envelope disassembly	2.50E-04				
12	glucose transport	2.50E-04				
12	hexose transport	2.50E-04				
12	nuclear pore complex assembly	1.64E-03				

<List of Pathways>

	**Liver of db/db mice**				**HepG2 treated with ESB**	
**Module**	**Pathway**	**FDR**		**Module**	**Pathway**	**FDR**
0	Proteasome	1.25E-04		0	Chronic myeloid leukemia	5.88E-05
0	NF-kB signaling pathway	1.14E-03		0	Platelet activation	6.67E-05
0	Neurotrophin signaling pathway	4.04E-03		0	Rap1 signaling pathway	7.14E-05
1	RIG-I-like receptor signaling pathway	1.11E-04		0	ErbB signaling pathway	9.52E-05
1	Autoimmune thyroid disease	1.25E-04		0	Proteoglycans in cancer	1.05E-04
1	Hematopoietic cell lineage	1.67E-04		0	T cell receptor signaling pathway	1.11E-04
1	Regulation of autophagy	1.82E-04		0	B cell receptor signaling pathway	1.25E-04
1	PI3K-Akt signaling pathway	2.00E-04		0	Osteoclast differentiation	1.82E-04
1	Prolactin signaling pathway	2.50E-04		0	Axon guidance	2.00E-04
1	Jak-STAT signaling pathway	5.00E-04		0	Colorectal cancer	2.61E-04
1	Cytokine-cytokine receptor interaction	5.00E-04		0	Leukocyte transendothelial migration	3.33E-04
1	Type II diabetes mellitus	5.39E-04		0	Fc epsilon RI signaling pathway	3.46E-04
1	Herpes simplex infection	9.33E-04		0	JAK-STAT signaling pathway	4.82E-04
1	Cytosolic DNA-sensing pathway	1.39E-03		0	Bacterial invasion of epithelial cells	5.00E-04
1	Measles	1.60E-03		0	Pathways in cancer	6.33E-04
1	Hepatitis C	1.63E-03		0	Measles	1.03E-03
1	Influenza A	3.71E-03		0	Natural killer cell mediated cytotoxicity	1.03E-03
1	Tuberculosis	3.92E-03		0	Insulin signaling pathway	1.18E-03
1	Toll-like receptor signaling pathway	5.73E-03		0	Chemokine signaling pathway	1.18E-03
1	Intestinal immune network for IgA production	6.72E-03		0	VEGF signaling pathway	1.23E-03
2	Fatty acid metabolism	1.11E-04		0	Renal cell carcinoma	1.70E-03
2	Fatty acid degradation	1.25E-04		0	Pancreatic cancer	1.70E-03
2	Thyroid hormone signaling pathway	1.43E-04		0	Melanoma	2.30E-03
2	Adipocytokine signaling pathway	1.67E-04		0	Cholinergic synapse	2.51E-03

2	Fatty acid biosynthesis	2.14E-04		0	Adherens junction	2.53E-03
2	PPAR signaling pathway	2.50E-04		0	Aldosterone-regulated sodium reabsorption	2.55E-03
3	Ras signaling pathway	5.00E-04		0	Regulation of actin cytoskeleton	2.57E-03
3	Chronic myeloid leukemia	9.50E-04		0	Neurotrophin signaling pathway	3.20E-03
3	T cell receptor signaling pathway	2.14E-03		0	Ras signaling pathway	3.26E-03
3	Neurotrophin signaling pathway	3.09E-03		0	Phosphatidylinositol signaling system	3.41E-03
3	Natural killer cell mediated cytotoxicity	4.44E-03		0	Carbohydrate digestion and absorption	3.46E-03
3	Glioma	6.78E-03		0	Type II diabetes mellitus	4.05E-03
3	Melanoma	8.47E-03		0	Progesterone-mediated oocyte maturation	4.80E-03
3	Prolactin signaling pathway	8.67E-03		0	Endometrial cancer	4.94E-03
7	Glycerophospholipid metabolism	1.25E-04		0	Fc gamma R-mediated phagocytosis	5.06E-03
7	Vascular smooth muscle contraction	1.67E-04		0	cAMP signaling pathway	5.86E-03
7	Arachidonic acid metabolism	2.00E-04		0	Non-small cell lung cancer	5.90E-03
7	Pancreatic secretion	2.00E-04		0	Acute myeloid leukemia	6.16E-03
7	Ras signaling pathway	2.50E-04		0	Focal adhesion	6.35E-03
7	Ether lipid metabolism	3.33E-04		0	Estrogen signaling pathway	6.42E-03
7	Linoleic acid metabolism	5.00E-04		0	Choline metabolism in cancer	6.42E-03
7	alpha-Linolenic acid metabolism	1.00E-03		0	mTOR signaling pathway	6.85E-03
7	MAPK signaling pathway	2.00E-03		0	Chagas disease (American trypanosomiasis)	7.22E-03
7	Fc gamma R-mediated phagocytosis	7.62E-03		0	HIF-1 signaling pathway	7.48E-03
7	Dorso-ventral axis formation	8.26E-03		0	Toll-like receptor signaling pathway	7.48E-03
7	Thyroid cancer	8.69E-03		0	Oxytocin signaling pathway	7.60E-03
7	Retrograde endocannabinoid signaling	8.89E-03		0	Glioma	8.23E-03
7	TNF signaling pathway	9.30E-03		0	Central carbon metabolism in cancer	8.92E-03
8	Protein processing in endoplasmic reticulum	1.00E-03		0	cGMP-PKG signaling pathway	9.06E-03
9	Ribosome	1.67E-04		1	Proteoglycans in cancer	7.69E-05
10	Ubiquitin mediated proteolysis	1.00E-03		1	Pathways in cancer	8.33E-05
10	Nucleotide excision repair	7.33E-03		1	HTLV-I infection	9.09E-05
12	RNA transport	1.67E-04		1	Melanogenesis	1.11E-04
				1	Hippo signaling pathway	1.43E-04
				1	Hedgehog signaling pathway	1.67E-04
				1	Signaling pathways regulating pluripotency of stem cells	2.00E-04
				1	Basal cell carcinoma	5.00E-04
				1	Wnt signaling pathway	1.00E-03
				2	Proteasome	5.00E-04
				5	Protein processing in endoplasmic reticulum	6.00E-03
				6	Vascular smooth muscle contraction	5.00E-04
				6	Ras signaling pathway	1.00E-03
				6	Linoleic acid metabolism	1.10E-03
				6	alpha-Linolenic acid metabolism	1.43E-03
				6	MAPK signaling pathway	1.60E-03
				6	Ether lipid metabolism	2.29E-03
				6	Hepatitis C	2.77E-03
				6	Arachidonic acid metabolism	4.86E-03
				6	Influenza A	4.86E-03
				6	Prolactin signaling pathway	4.86E-03
				6	Pancreatic cancer	4.88E-03
				6	Leishmaniasis	4.93E-03
				6	Fc gamma R-mediated phagocytosis	6.38E-03
				6	Glycerophospholipid metabolism	6.73E-03
				6	Toll-like receptor signaling pathway	7.79E-03
				6	Dorso-ventral axis formation	8.22E-03
				6	Thyroid cancer	8.70E-03
				6	Toxoplasmosis	8.76E-03
				6	Thyroid hormone signaling pathway	8.76E-03
				7	Fructose and mannose metabolism	7.69E-05
				7	Non-alcoholic fatty liver disease (NAFLD)	9.09E-05
				7	Hypertrophic cardiomyopathy (HCM)	1.00E-04
				7	Circadian rhythm	1.67E-04
				7	Oxytocin signaling pathway	2.00E-04
				7	Insulin signaling pathway	2.50E-04
				7	FoxO signaling pathway	3.33E-04
				7	AMPK signaling pathway	5.00E-04
				7	Adipocytokine signaling pathway	5.00E-04
				7	Galactose metabolism	9.41E-04
				7	HIF-1 signaling pathway	1.32E-03
				7	Carbohydrate digestion and absorption	1.91E-03
				7	Starch and sucrose metabolism	3.27E-03
				7	Glycolysis / Gluconeogenesis	4.83E-03

				7	PI3K-Akt signaling pathway	8.33E-03
				8	DNA replication	1.00E-04
				8	Homologous recombination	1.43E-04
				8	Nucleotide excision repair	2.00E-04
				8	Mismatch repair	3.85E-04
				8	Fanconi anemia pathway	1.00E-03
				10	Endocytosis	3.33E-04
				12	Spliceosome	5.00E-04

Supplementary Fig. S1. is moved to the last part of the supplementary figures.

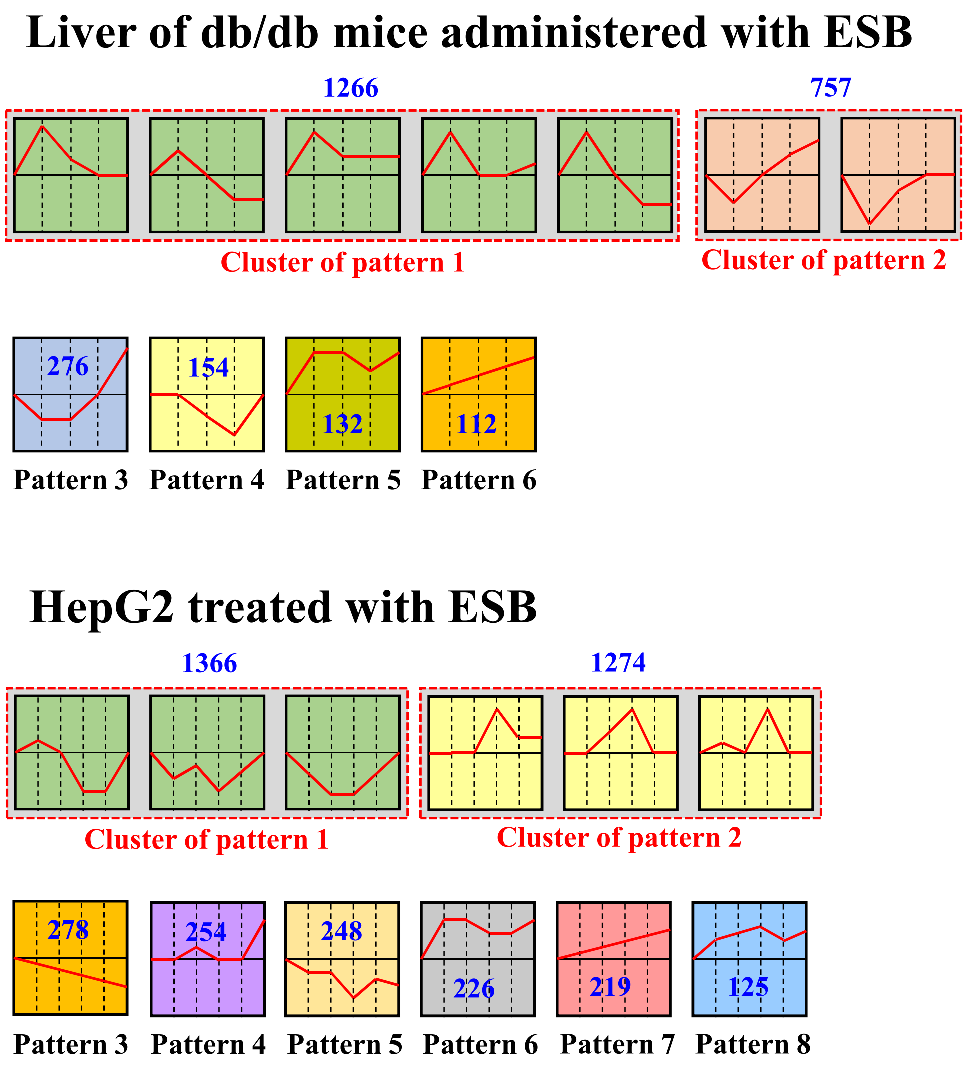

Supplementary Fig. S2. Significant patterns (FDR < 0.001) of gene expression analyzed by STEM. The numbers represent numbers of genes included in each patterns. Two major patterns with the largest numbers of genes are colored in red.

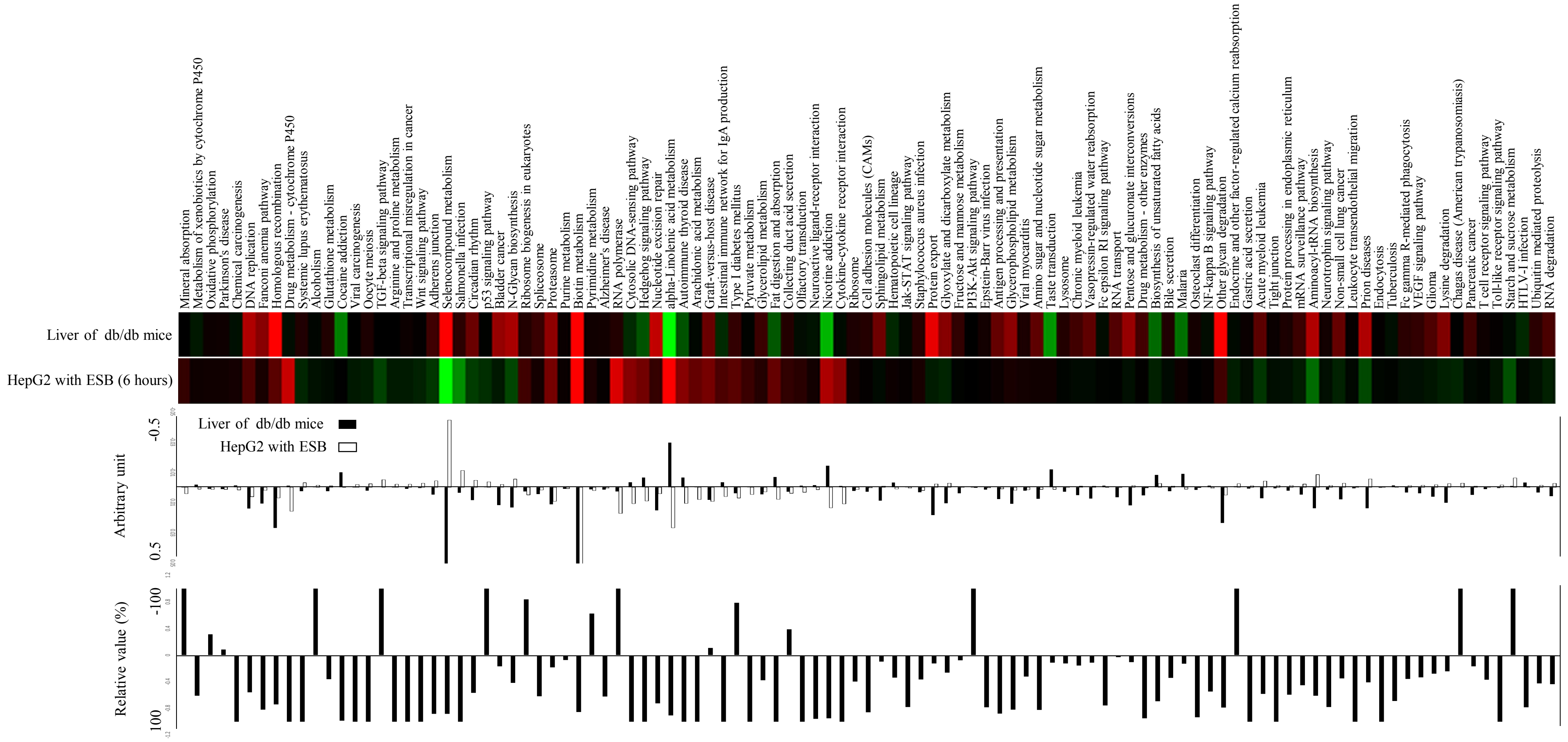

Supplementary Fig. S3. Pathway activities were compared between db/db mouse liver and HepG2 cells treated with ESB for 6 hours. The relative values for the pathway activities of ESB-treated HepG2 cells relative to those in db/db mouse liver are also shown.

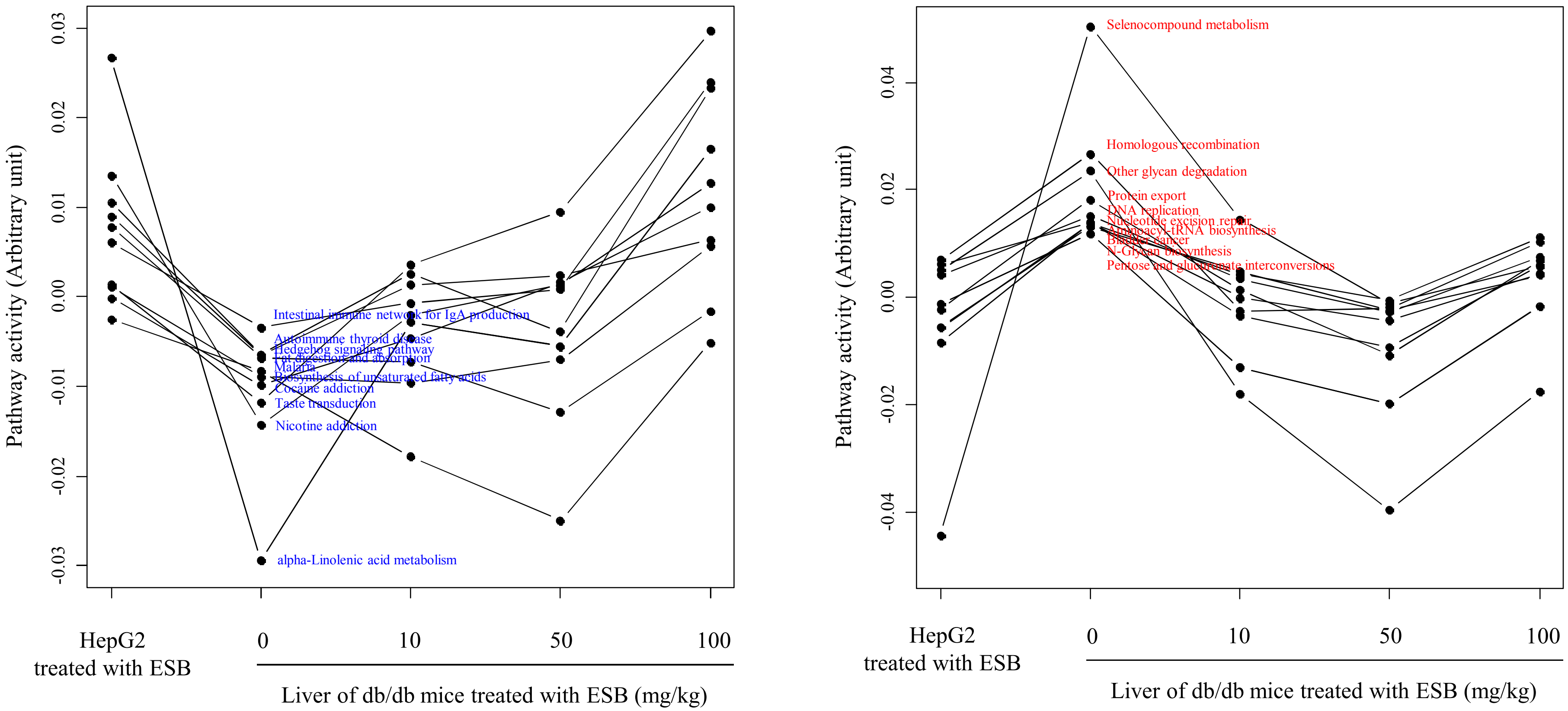

Supplementary Fig. S4. Top 10 pathways recovered by ESB in db/db mouse liver. The top 10 pathways most changed in db/db mouse liver were plotted. The downregulated pathways in db/db mouse liver were alpha-Linolenic acid metabolism, Nicotine addiction, Taste transduction, Cocaine addiction, Malaria, Biosynthesis of unsaturated fatty acids, Fat digestion and absorption, Hedgehog signaling pathway, Autoimmune thyroid disease, and Intestinal immune network for IgA production. The upregulated pathways in db/db mouse liver were Pentose and glucuronate interconversions, N-Glycan biosynthesis, Bladder cancer, Aminoacyl-tRNA biosynthesis, DNA replication, Nucleotide excision repair, Protein export, Other glycan degradation, Homologous recombination, and Selenocompound metabolism.

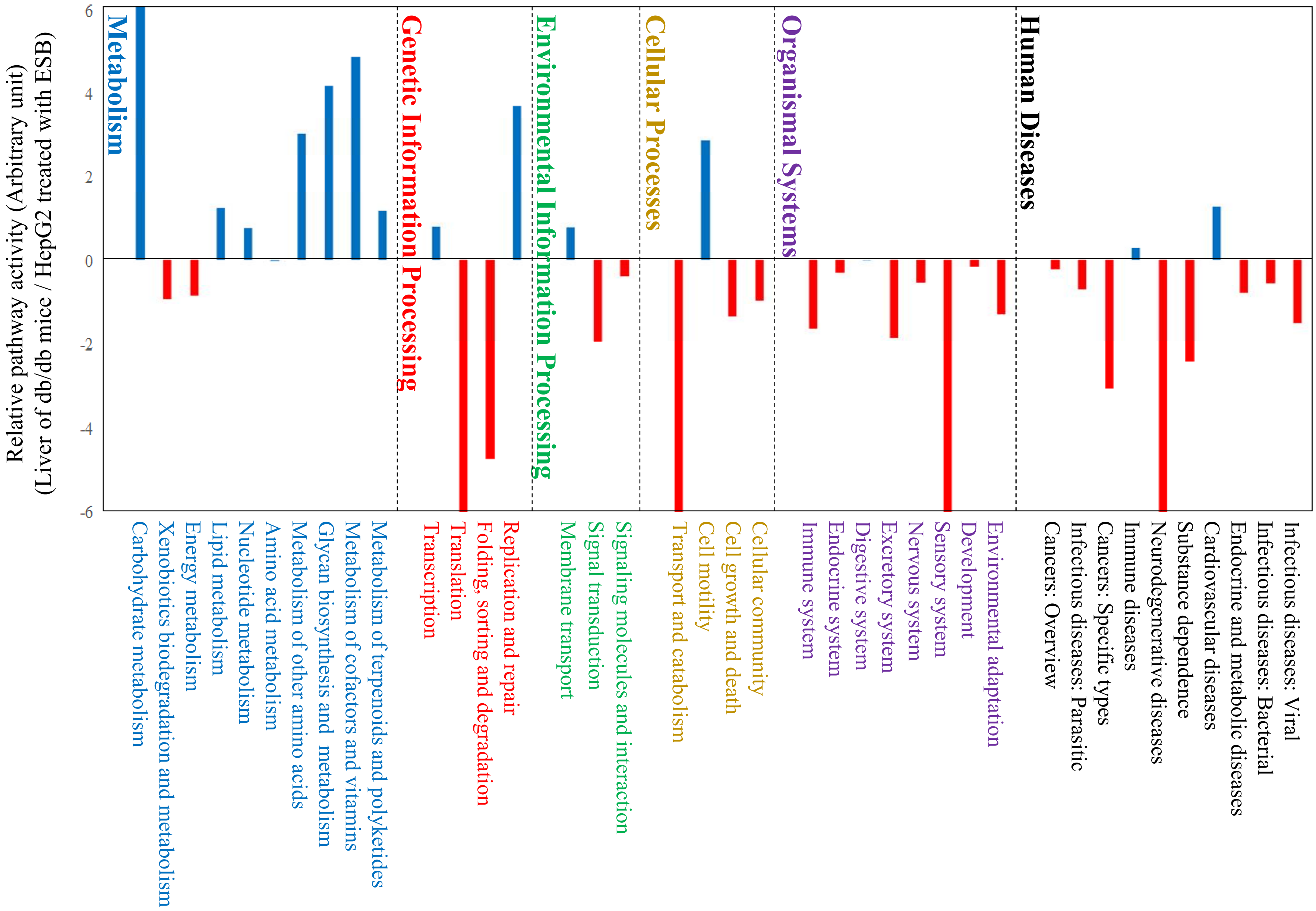

Supplementary Fig. S5. Functional distribution of genes in db/db mouse liver and HepG2 cells treated with ESB. The expression values of genes compared with the normal control in each functional category were log transformed and then linearly combined with a weight of –1 for genes acting as repressors. The measured values from db/db mouse liver were divided by those from HepG2 cells treated with ESB. Statistical significance was measured by random permutation of genes (n = 1000). The information for functional categories was imported from KEGG (http://www.genome.jp/kegg/).

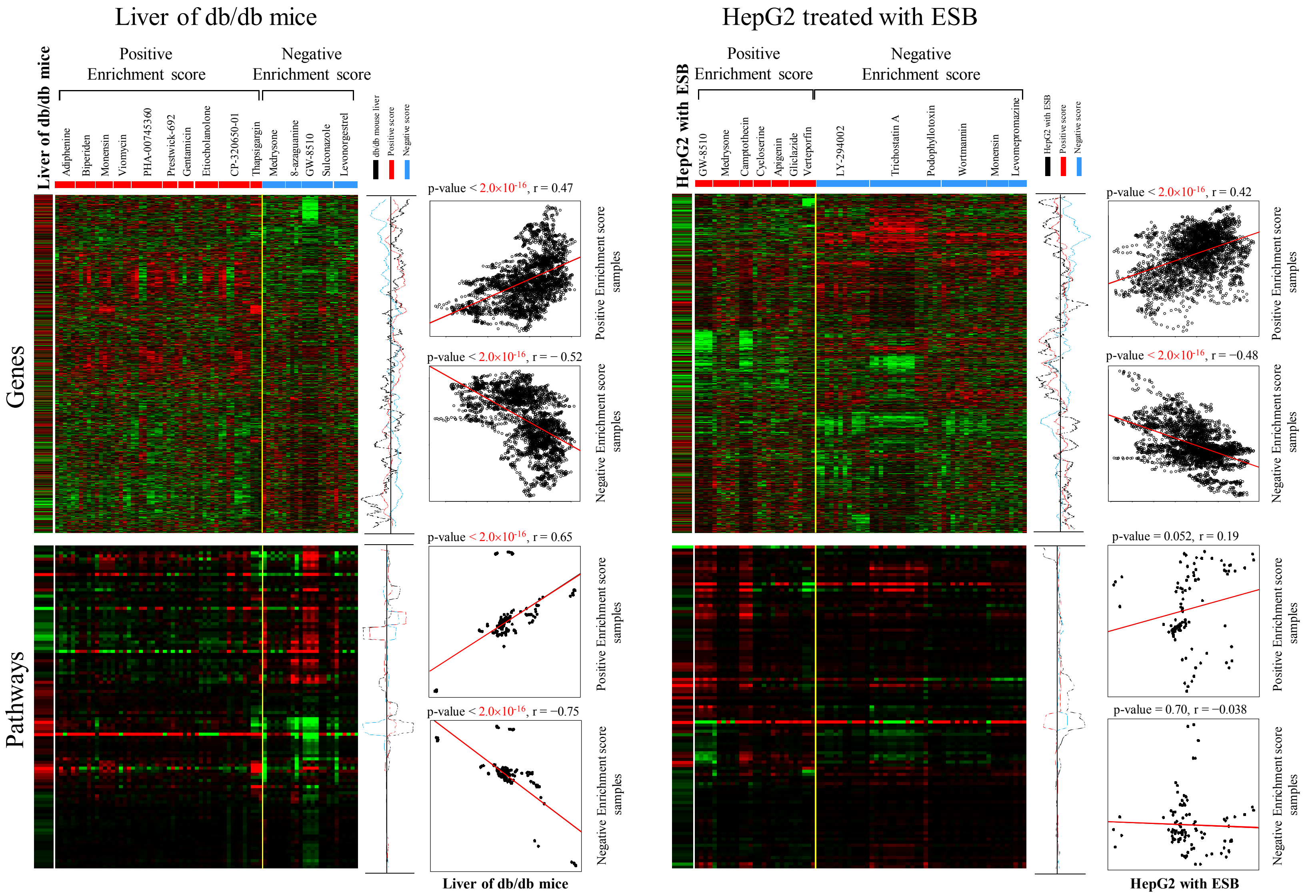

Supplementary Fig. S6. Comparison of gene expression values (or activity values) from db/db mouse liver with an averaged value of gene expression (or activity values) from highly ranked (permutated p-value < 0.001) cmap samples of positive or negative enrichment scores shows positive ($r = 0.47$, $p < 0.01$ for gene expression and $r = 0.65$, $p < 0.01$ for pathway activity) or negative ($r = -0.52$, $p < 0.01$ for gene expression and $r = -0.75$, $p < 0.01$ for pathway activity) correlation, respectively. In the case of ESB-treated HepG2 cells, we also measured similar results in gene expression pattern. Positive ($r = 0.42$, $p < 0.01$) or negative ($r = -0.48$, $p < 0.01$) correlation was observed between gene expression values of ESB-treated HepG2 cells and those of averaged samples with positive or negative enrichment scores in the cmap database, respectively. However, in the pathway activity of ESB-treated HepG2 cells, no statistically significant relationship was found.

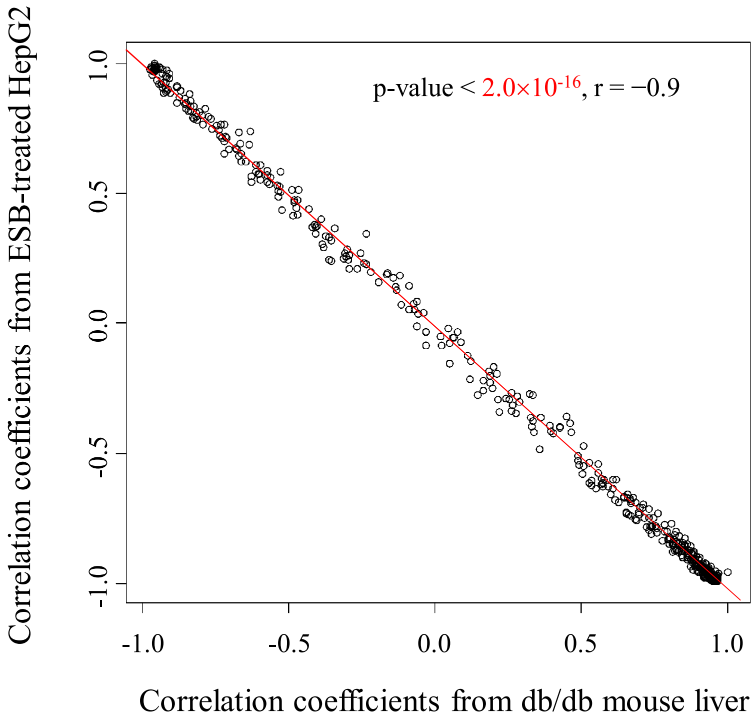

Supplementary Fig. S7. Validation of the cmap-based approach. Five hundred randomly chosen chemicals from the cmap database were analysed by cmap. The distribution of enrichment scores for random chemicals was compared with that of db/db mouse liver and ESB-treated HepG2 cells. Correlation coefficients obtained from db/db mouse liver and ESB-treated HepG2 cells were plotted for random chemicals.

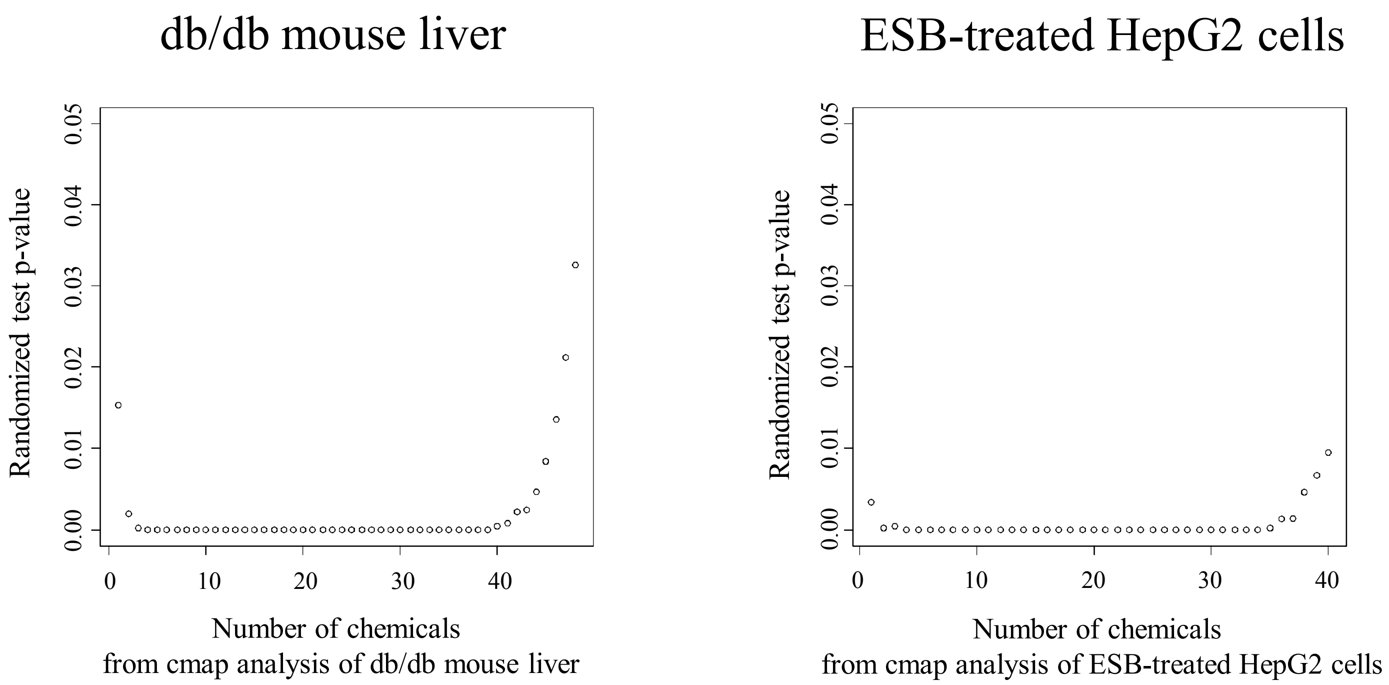

Supplementary Fig. S8. Keyword-based literature-mining analysis of cmap results. We used all the chemicals (1309 chemicals) present in the cmap database to search for papers with keywords connected to diabetes-related diseases and conditions. We then changed the referred numbers of papers to 0 (absence of reference paper) and 1 (presence of reference paper) to eliminate bias induced by a few heavily referred chemicals. We then compared the total sum of the original dataset with the same-sized randomly selected dataset repeatedly 1000 times. The relative positon of the sum of the original dataset among those of the randomized test sets was measured as a ratio and used as a statistic (randomized test p-value). We measured this value by increasing the number of chemicals included in the original dataset starting from the most referred chemical.

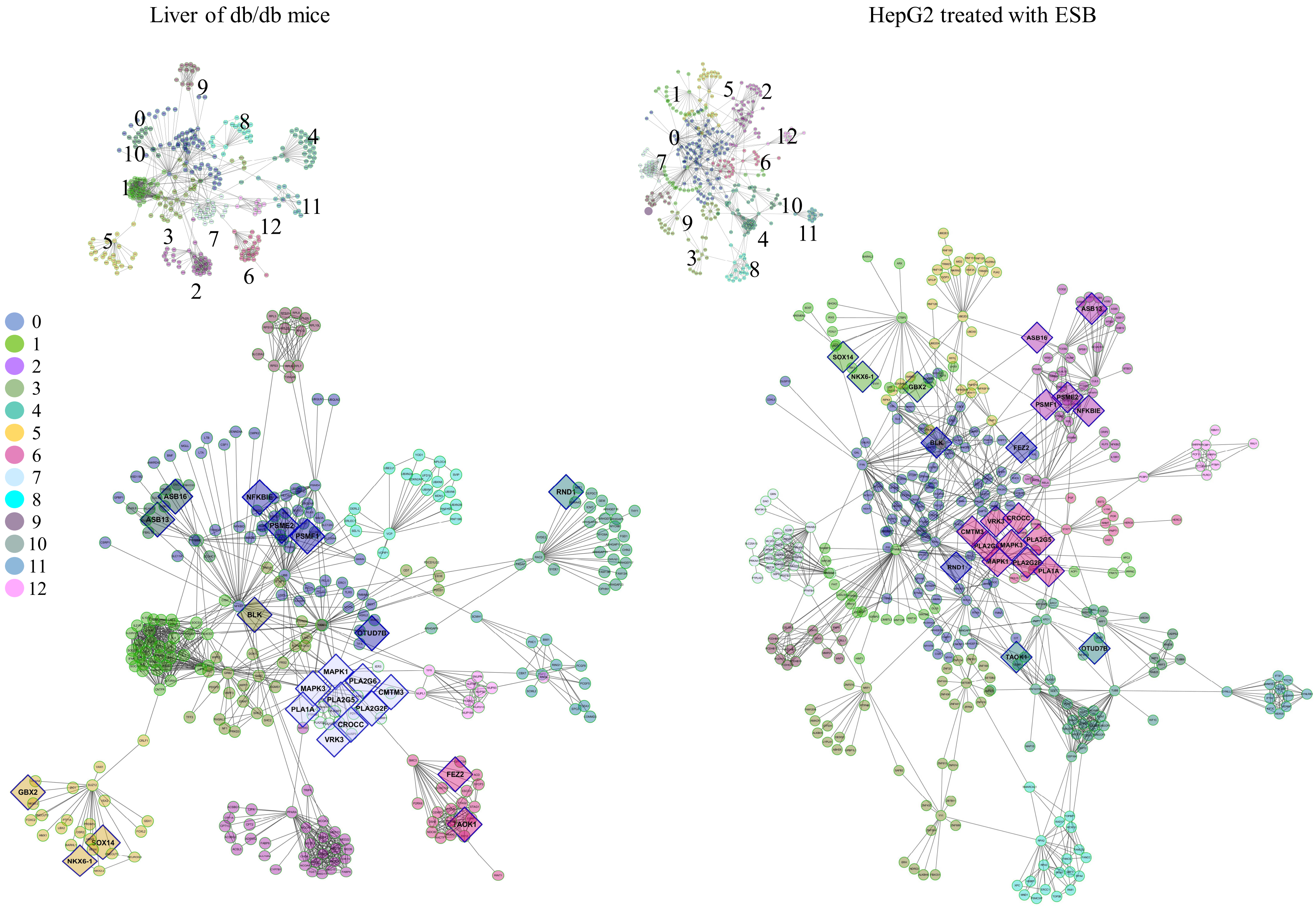

Supplementary Fig. S9. Common genes in the functional networks of db/db mouse liver and HepG2 cells treated with ESB are highlighted in with a diamond shape.

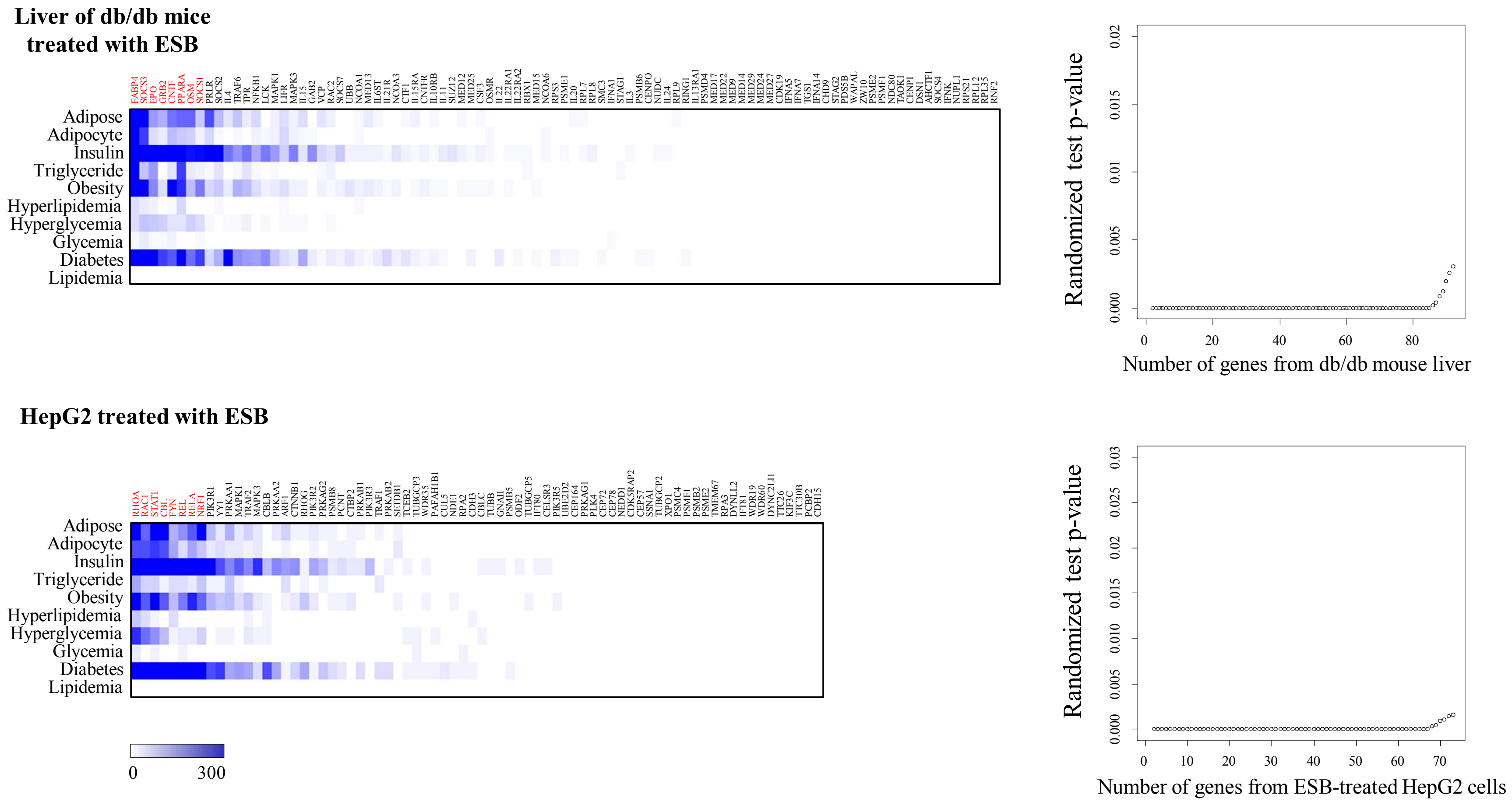

Supplementary Fig. S10. A literature-mining approach was used to measure the association between core genes in the functional network analysis and diabetes-related diseases. For the keywords of genes, the core node genes (>10 degrees) were used. The numbers of papers having keywords in the title or abstract published in 1990–2016 were measured in the PubMed database using the PubMatrix algorithm [47]. The numbers of papers are indicated by different colours on a scale bar. For the random permutation-based test of the resultant number of papers, we used randomly selected genes to search for papers with keywords for diabetes-related diseases and conditions. Then we changed the referred numbers of papers into 0 (absence of reference) and 1 (presence of reference) to eliminate bias induced by a few heavily referred chemicals. We then compared the total sum of the original dataset with the same-sized randomly selected dataset repeatedly 1000 times. The relative positon of the sum of the original dataset among those of the randomized test sets was measured as a ratio and used as a statistic (randomized test p-value). We measured this value by increasing the number of genes included in the original dataset starting from the most referred gene.

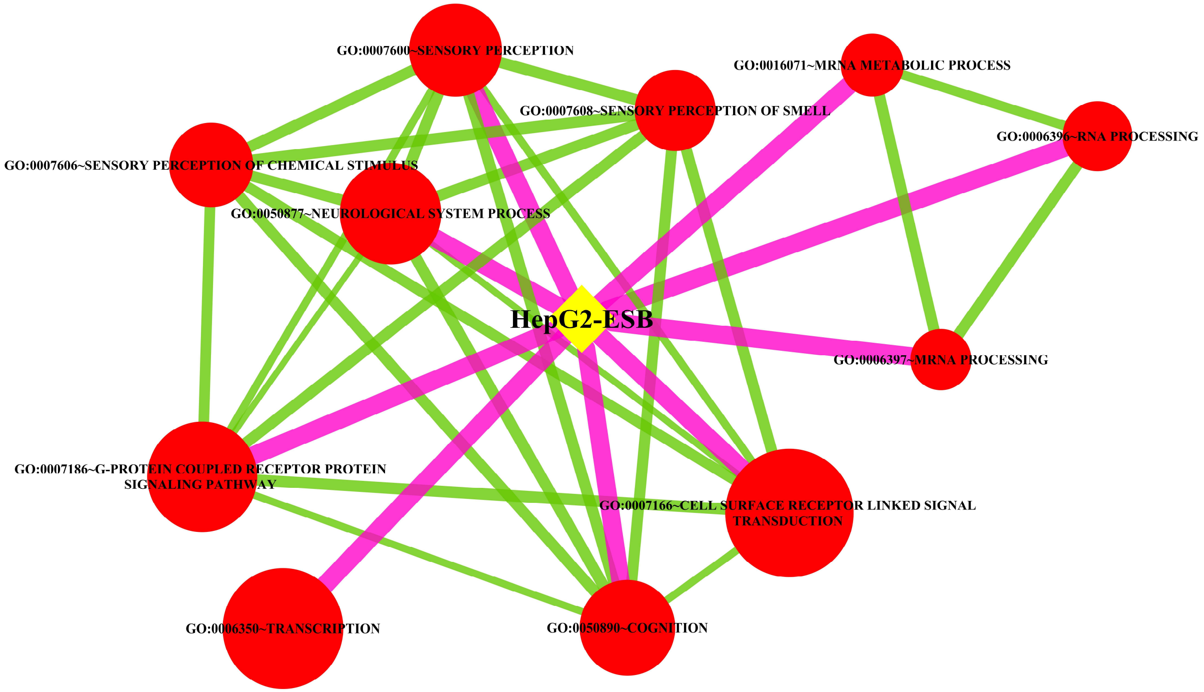

Supplementary Fig. S11. Enrichment map analysis. GO terms significantly associated with db/db mouse liver (FDR < 0.01) were used to construct the GO term network by implementing the Enrichment map program. The size of each node represents the number of genes included in that category of GO term, and the thickness of each edge line represents the number of common genes between two GO terms. Differentially expressed genes in HepG2 cells after ESB treatment for 6 hours are mapped on this GO term network. The relationship of GO terms with differentially expressed genes in HepG2 cells after ESB treatment was obtained using a hypergeometric test with 0.01 cut-off for the determination of edge weight. Yellow diamonds represent genes from ESB-treated HepG2 cells and purple edge lines show the presence of ESB-responsive genes in HepG2 cells included in each GO term category. The thickness of each purple edge line is in proportion to the number of common genes.

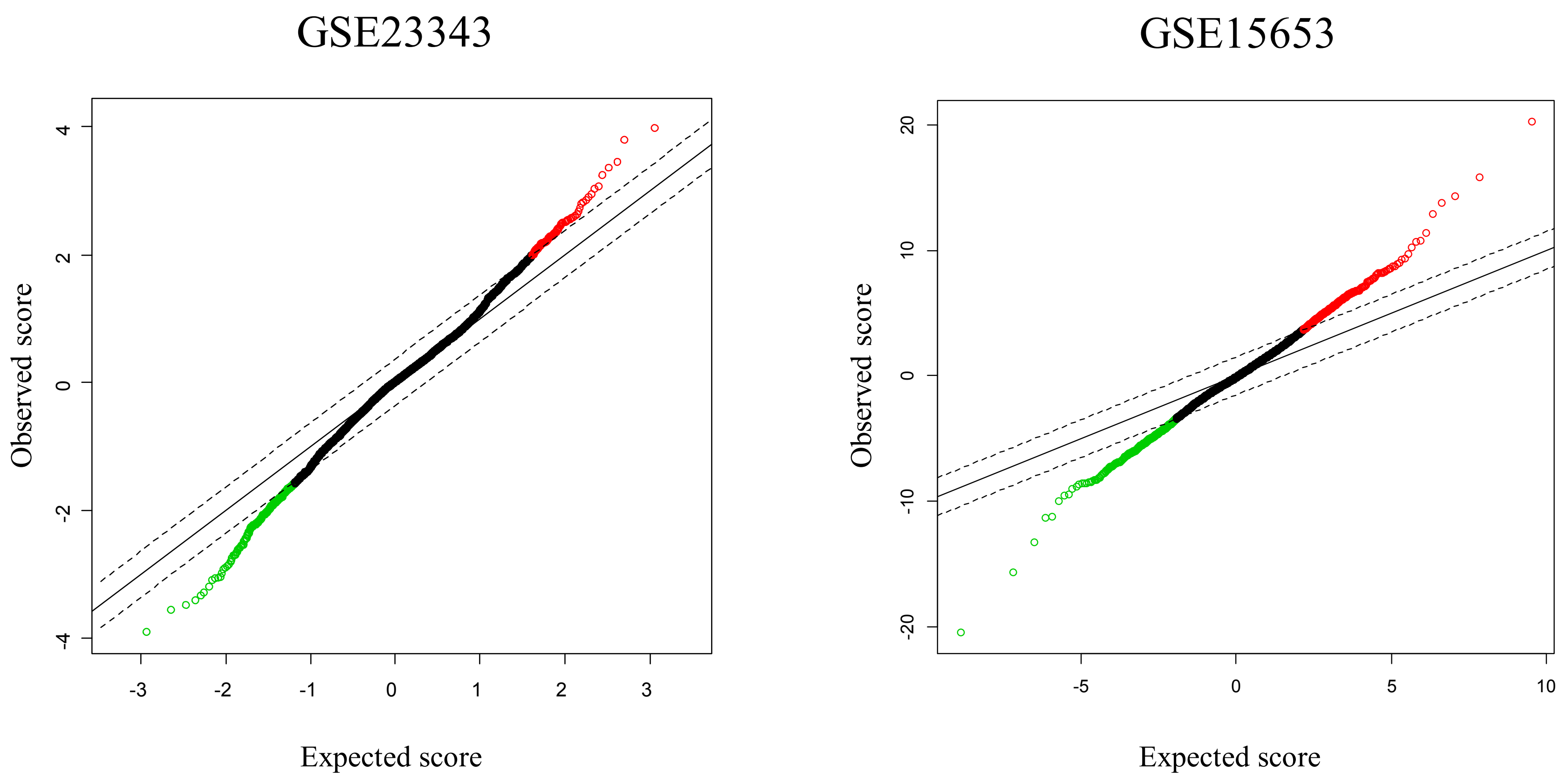

Supplementary Fig. S12. Genes used as input tags for cmap analysis were identified using Significance Analysis of Microarray (SAM). For the microarray data from the two public databases GSE23323 and GSE15653, SAM scores for the absolute values of 2.0 and 4.0 were used for the threshold value and corresponded to an FDR < 0.3 and FDR < 0.05, respectively. Red and green circles represent genes in the disease samples that were up- and downregulated, respectively, compared with normal control samples. The dotted line represents the threshold line.

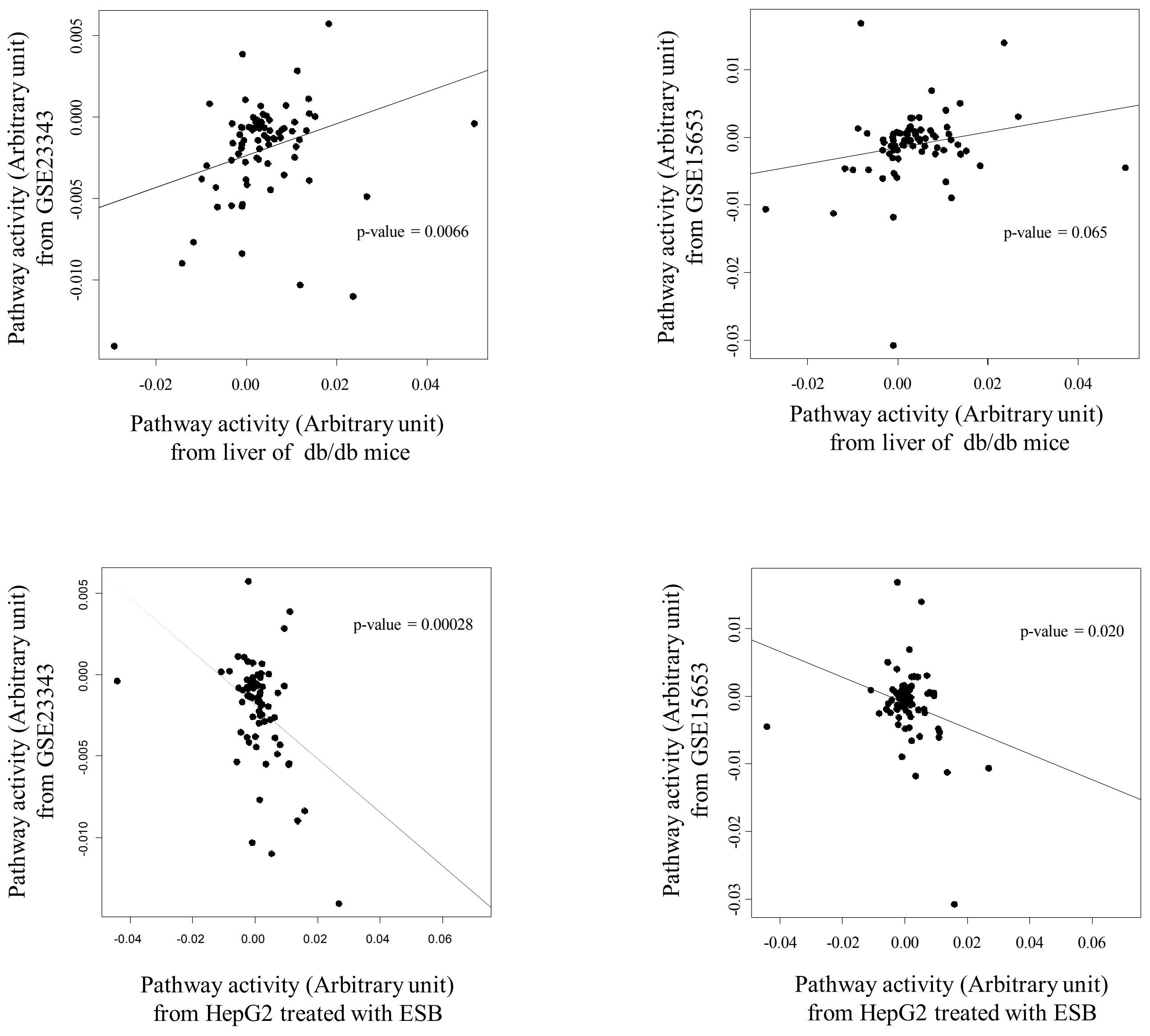

Supplementary Fig. S13. Comparison of pathway activities between db/db mouse liver, ESB-treated HepG2 cells, and liver samples of diabetic patients. Pathway activities from two publically available microarray databased of liver samples from diabetic patients (GSE23323 and GSE15653) were compared with those from db/db mouse liver and ESB-treated HepG2 cells.

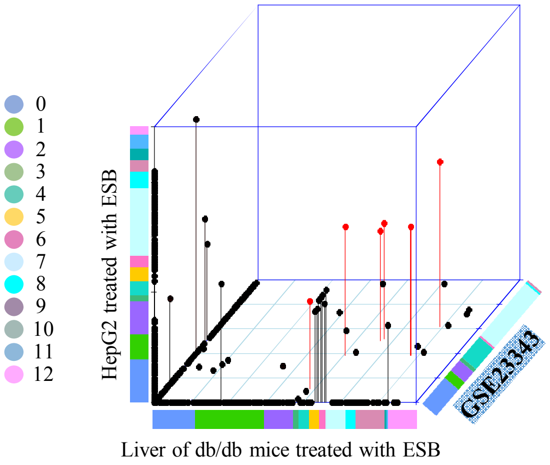

GO Terms	Module		
	db/db mice	GSE23343	HepG2
Phosphatidylcholine acyl-chain remodeling	7	4	6
Phosphatidylethanolamine acyl-chain remodeling	7	4	6
Small GTPase mediated signal transduction	4	3	0
Small molecule metabolic process	12	7	7
Transcription, DNA-dependent	5,11	6	3

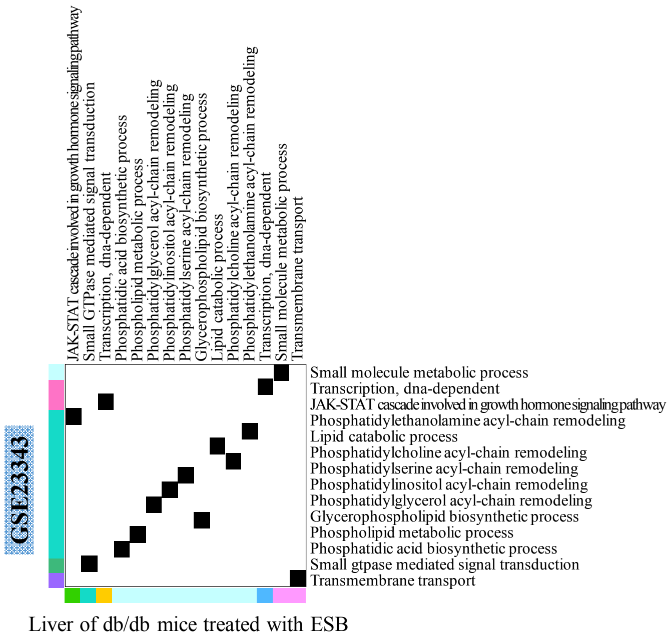

Supplementary Fig. S14. The functional interaction network was constructed from the gene expression patterns in db/db mouse liver, ESB-treated HepG2 cells, and liver samples from patients with diabetes (GSE23323 and GSE15653) by implementing the Reactome FI application. Common GO terms associated with each module ($p < 0.01$) are shown in red and listed. Functional similarities between the db/db mouse liver and human liver samples (GSE23323 and GSE15653) are also plotted according to the GO terms associated with the modules.

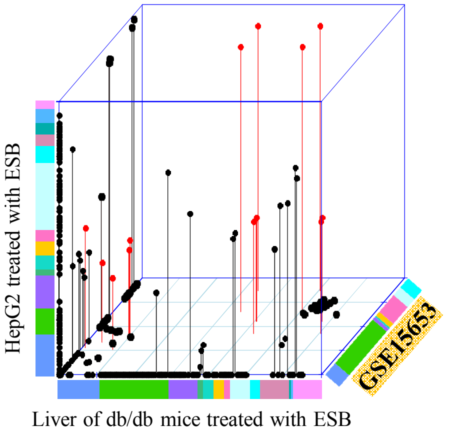

GO Terms	Module		
	db/db mice	GSE15653	HepG2
DNA damage response, signal transduction by p53 class mediator resulting in cell cycle arrest	0	1	2
Gene expression	2,9	6,8	12
Mitotic cell cycle	6,12	1	4
Negative regulation of ubiquitin-protein ligase activity involved in mitotic cell cycle	0	2	1
Positive regulation of NF-kB transcription factor activity	0	1	5
Positive regulation of ubiquitin-protein ligase activity involved in mitotic cell cycle	0	1	2
Protein poly ubiquitination	0	1	2
Transcription, DNA-dependent	5,11	2	3

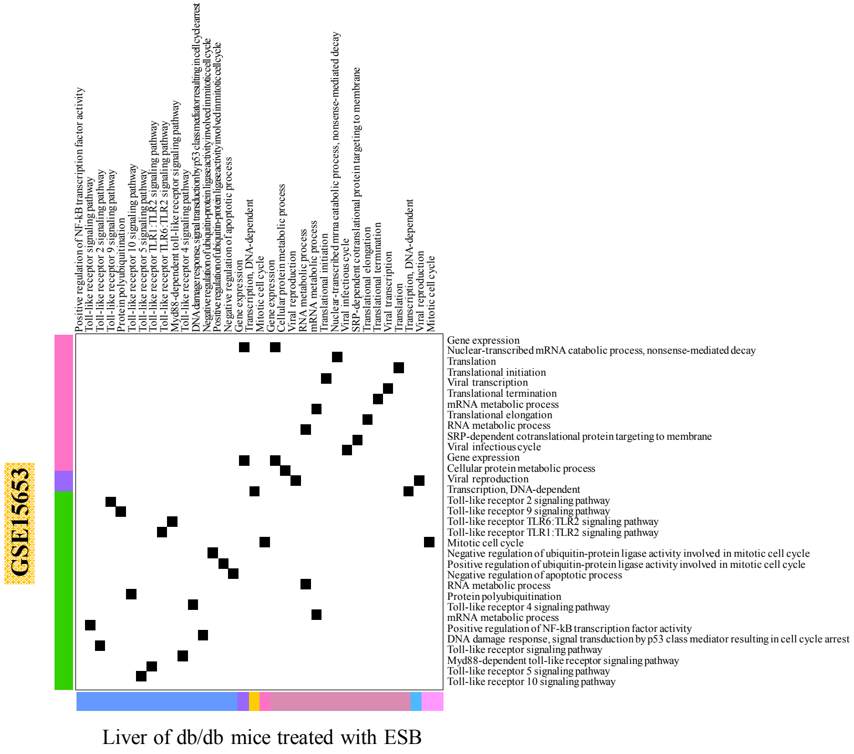

Supplementary Fig. S1. All gene lists about expression data of Fig. 3A.

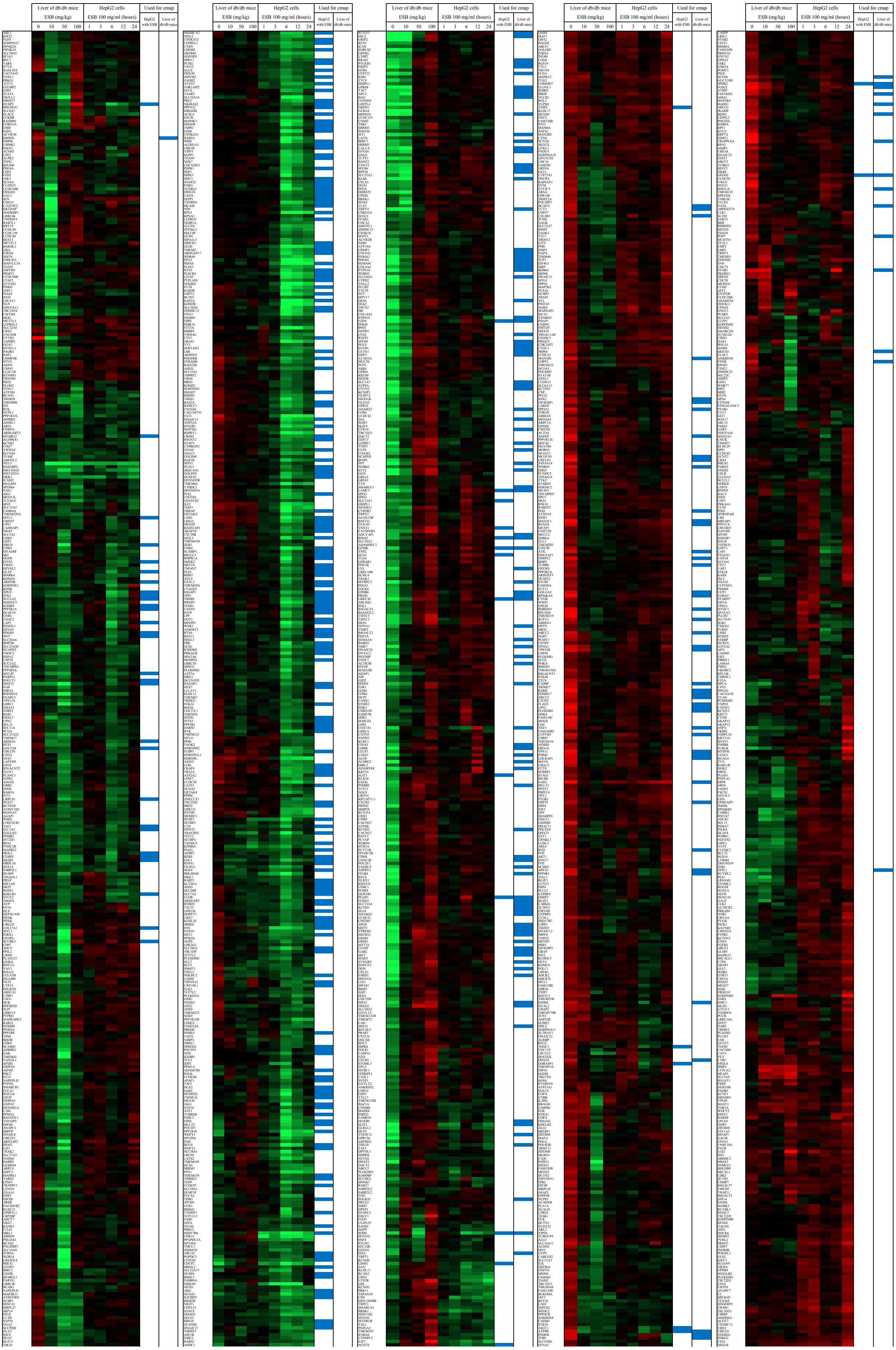

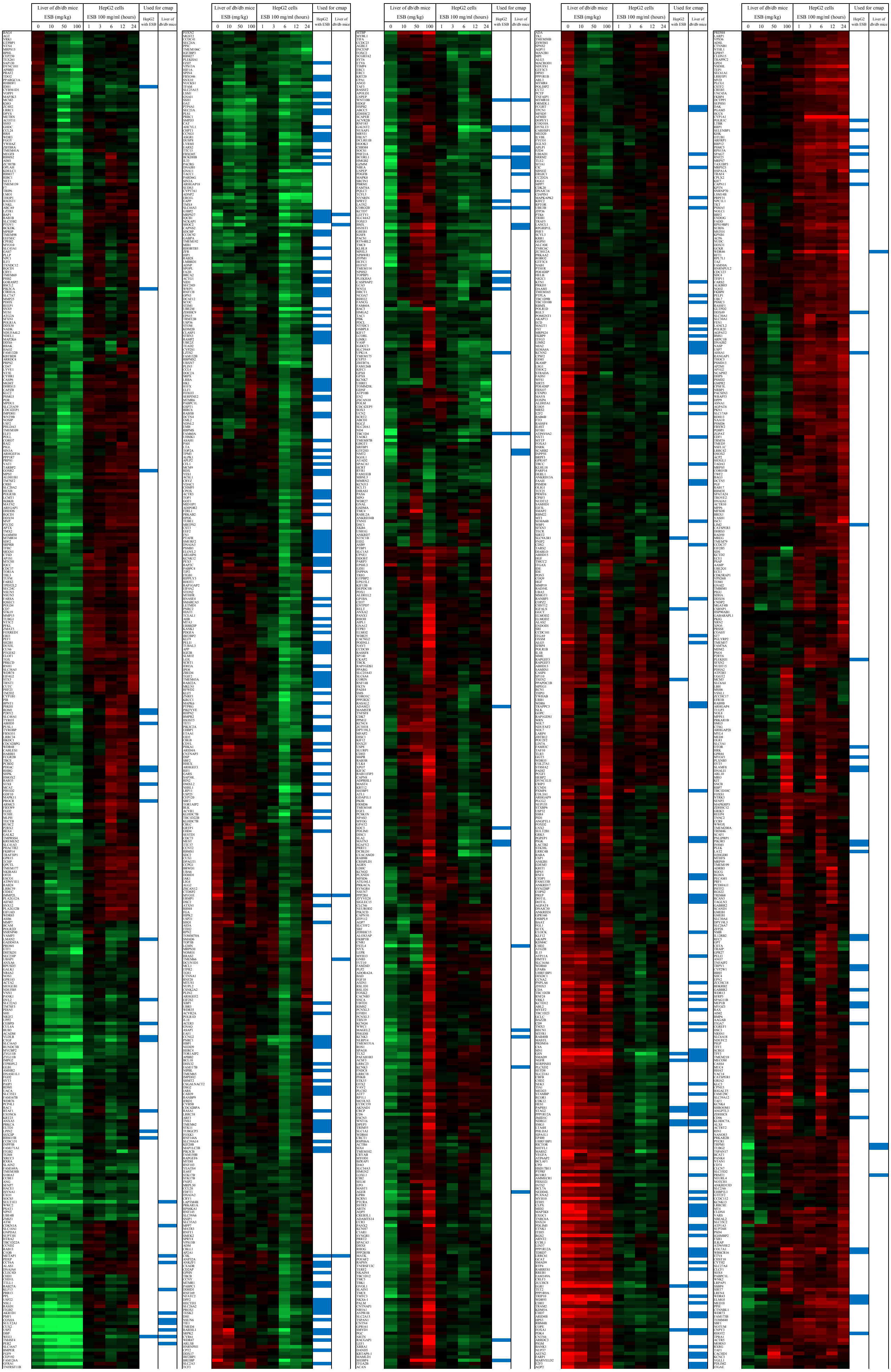
Liver of db/db mice
ESB (mg/kg)
0 10 50 100
HepG2 cells
ESB 100 mg/ml (hours)
1 3 6 12 24
Used for cmap
HepG2 with ESB
Liver of db/db mice

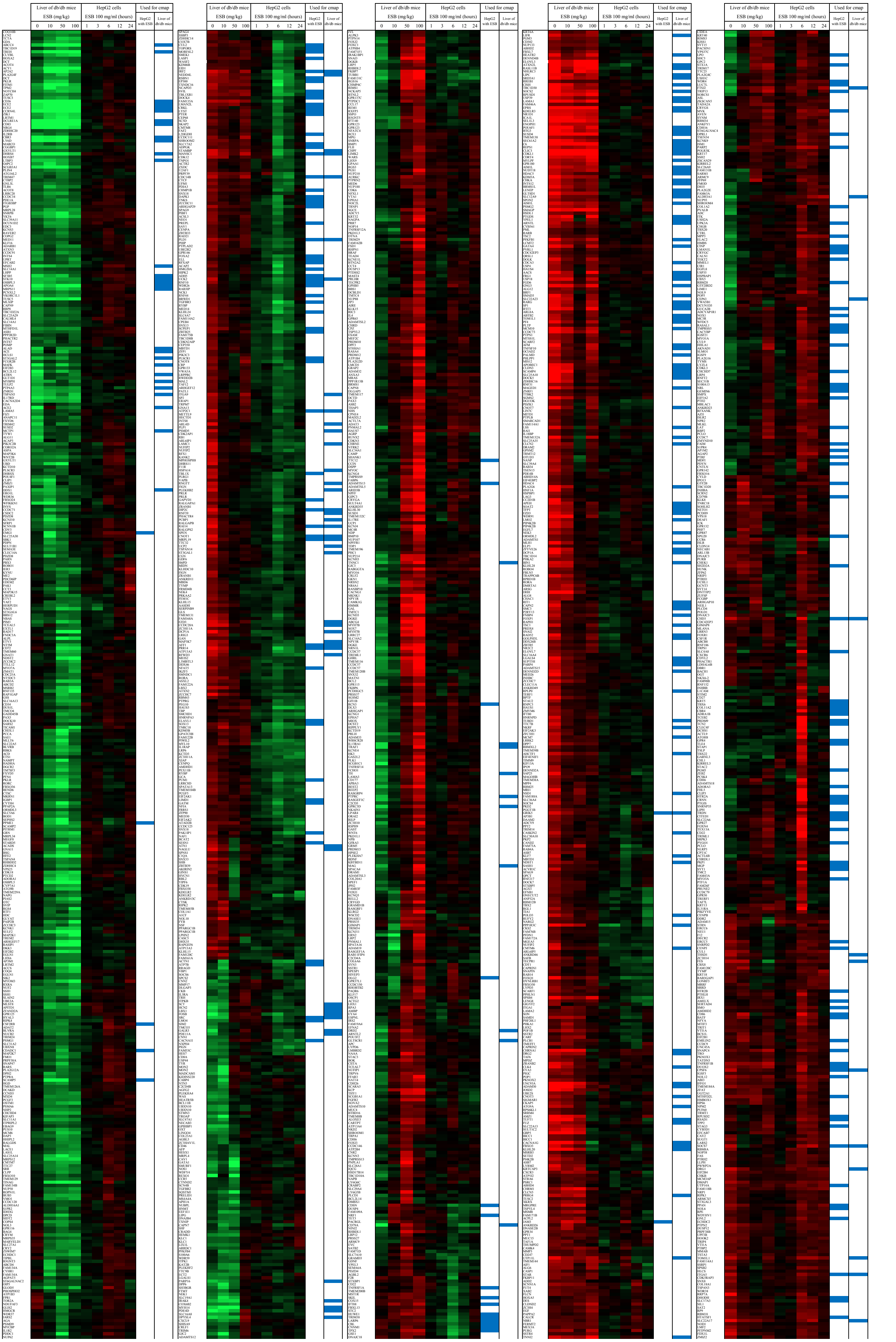
Liver of db/db mice
ESB (mg/kg)
0 10 50 100
HepG2 cells
ESB 100 mg/ml (hours)
1 3 6 12 24
Used for cmap
HepG2 with ESB
Liver of db/db mice

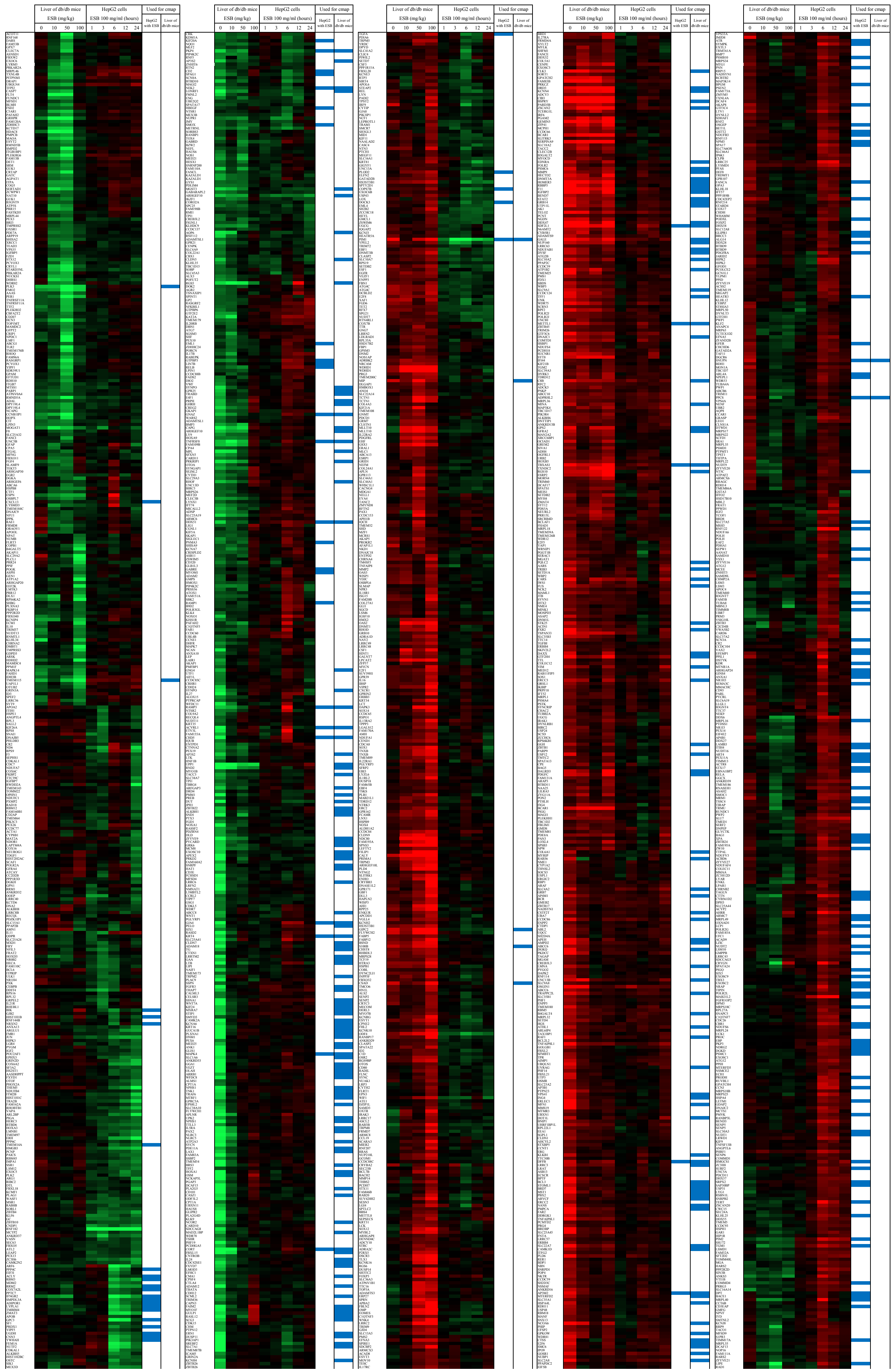
Liver of db/db mice
ESB (mg/kg)
0 10 50 100
HepG2 cells
ESB 100 mg/ml (hours)
1 3 6 12 24
Used for cmap
HepG2 with ESB
Liver of db/db mice

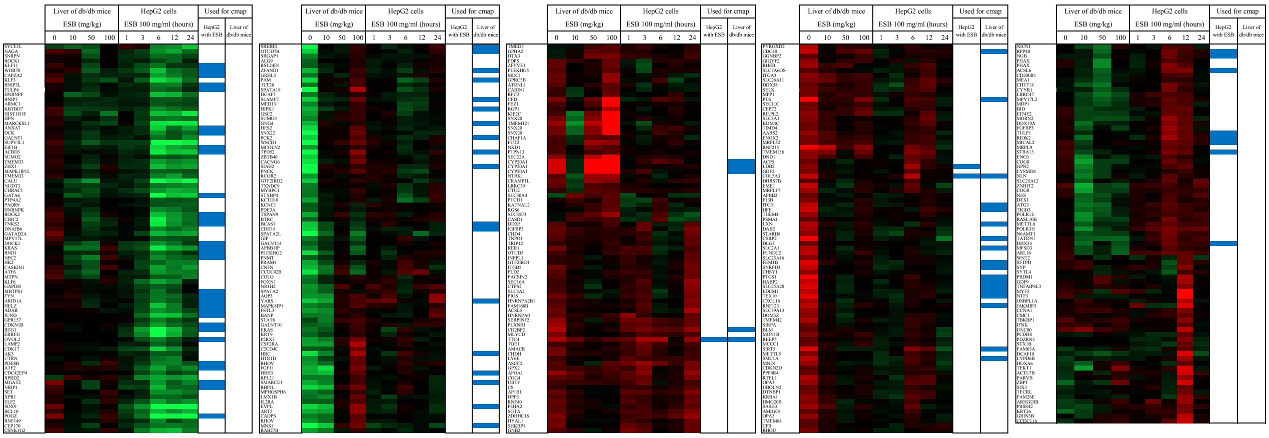
Liver of db/db mice
ESB (mg/kg)
0 10 50 100
HepG2 cells
ESB 100 mg/ml (hours)
1 3 6 12 24
Used for cmap
HepG2 with ESB
Liver of db/db mice
